# Peptide-Based Nanoassemblies in Gene Therapy and Diagnosis: Paving the Way for Clinical Application

**DOI:** 10.3390/molecules25153482

**Published:** 2020-07-31

**Authors:** Shabnam Tarvirdipour, Xinan Huang, Voichita Mihali, Cora-Ann Schoenenberger, Cornelia G. Palivan

**Affiliations:** 1 Department of Chemistry, University of Basel, Mattenstrasse 24a, 4058 Basel, Switzerland; shabnam.tarvirdipour@unibas.ch (S.T.); xinan.huang@unibas.ch (X.H.); voichita.mihali@unibas.ch (V.M.); 2 Department of Biosystem Science and Engineering, ETH Zurich, Mattenstrasse 26, 4058 Basel, Switzerland

**Keywords:** peptide nanoassemblies, micelles, nanovesicles, nanotubes, nanofibrils, nanoprobes, peptide conjugates, cell penetrating peptides, non-viral gene delivery, diagnostic imaging

## Abstract

Nanotechnology approaches play an important role in developing novel and efficient carriers for biomedical applications. Peptides are particularly appealing to generate such nanocarriers because they can be rationally designed to serve as building blocks for self-assembling nanoscale structures with great potential as therapeutic or diagnostic delivery vehicles. In this review, we describe peptide-based nanoassemblies and highlight features that make them particularly attractive for the delivery of nucleic acids to host cells or improve the specificity and sensitivity of probes in diagnostic imaging. We outline the current state in the design of peptides and peptide-conjugates and the paradigms of their self-assembly into well-defined nanostructures, as well as the co-assembly of nucleic acids to form less structured nanoparticles. Various recent examples of engineered peptides and peptide-conjugates promoting self-assembly and providing the structures with wanted functionalities are presented. The advantages of peptides are not only their biocompatibility and biodegradability, but the possibility of sheer limitless combinations and modifications of amino acid residues to induce the assembly of modular, multiplexed delivery systems. Moreover, functions that nature encoded in peptides, such as their ability to target molecular recognition sites, can be emulated repeatedly in nanoassemblies. Finally, we present recent examples where self-assembled peptide-based assemblies with “smart” activity are used in vivo. Gene delivery and diagnostic imaging in mouse tumor models exemplify the great potential of peptide nanoassemblies for future clinical applications.

## 1. Introduction

Peptides have several advantages that make them highly attractive building blocks for tailoring nanoassemblies to biomedical applications [1,2,3]. In this regard, the side chain properties of amino acids, including hydrophilicity, hydrophobicity, charge, and polarity, allow for accomplishing certain functions in biological systems [4]. In addition, a family of membrane active peptides are inherently capable of conferring targeting or membrane translocating properties upon different bioactive cargoes [2,5]. Playing a major role as modulator of many cellular functions [6], peptides have the potential to fulfil critical prerequisites for successful gene delivery and diagnostic systems [2,5]. There is increasing evidence for the incorporation of peptides as recognition/targeting moieties, gene delivery, and diagnosis modules in a broad range of biomedical applications [7].

Self-assembly of peptides into supramolecular structures takes place when adequate non-covalent interactions between peptide chains occur. Several other factors, including concentration, buffer composition (pH, ionic strength), and temperature also influence the self-assembly process [8]. Significant progress has been made in the field of self-assembly and a large variety of peptide-based supramolecular structures with customized properties were developed [4,9]. Consequently, the ease of “bottom-up” fabrication through rational design of amino acid sequences offers infinite smart nanoscale architectures, with sizes ranging from few to hundreds of nanometers [10].

In this review, we introduce different peptide-based nanoassemblies, such as micelles, vesicles, nanofibers, nanotubes, and nanoparticles, with a multitude of distinct physicochemical properties. We highlight the principles that lead to the intended supramolecular structure and how they are tailored to carry out specific functions. Due to the versatility of peptides and the vast diversity of the ensuing nanoassemblies, we chose to focus on two applications that are of utmost medical relevance, namely gene delivery and diagnosis. The prospect of combining the advantageous properties of peptides with those of dimensions in the nanoscale regime make peptide-based nanomaterials prime candidates for many biomedical applications, in particular as vehicles for carrying therapeutic agents like nucleic acids or detectable probes to specific sites in the body [11]. Therefore, we look into the potential of peptide-based nanoassemblies as delivery vector in gene therapy because of their ability to bind/conjugate or entrap/condense DNA or RNA, and in diagnostic imaging, because of their targeting propensity [12].

In recent years, gene insertion and interference therapies using viral vectors have made a ground breaking impact in the treatment of rare inherited diseases, neurological disorders, cardiac diseases, and cancer [13]. Several disadvantages of viral-vectors, such as high toxicity and immunogenicity, limitation in size of transgenic DNA, and high manufacturing cost, have triggered the rapid expansion of non-viral delivery systems, including peptide-based vectors [14]. Thus, we will discuss peptide-based assemblies with regard to the advantages they offer for increasing the efficacy of gene delivery in vivo, such as targeting and overcoming different kinds of biological barriers like plasma and endosomal membranes [14].

The sensitive and accurate detection of specific targets in the body, in particular molecular markers of diseases in organs and tissues, is key to diagnostic imaging. Corresponding nanoprobes that enable a rapid and accurate diagnosis are of crucial importance for effective therapy and the prevention of disease progression to ultimately incurable stages [15]. Clinical and preclinical applications of wide range of imaging nanoprobes, emphasize the potential offered by combining modern nanotechnology and molecular imaging science for early-stage diagnosis [16,17,18]. In recent years, peptide-based nanoassemblies have made considerable headway towards providing novel advanced molecular *imaging nanoprobes* with great accuracy and precision [19]. In modern imaging, peptide-based nanoprobes aimed at improving in vitro and in vivo performance of water-soluble molecular probes [19,20]. Conclusively, we describe some recent in vivo examples of diagnostic imaging and gene delivery that reveal the great potential of peptide-based nanoassemblies, for future clinical applications.

## 2. Peptide-Based Supramolecular Nanoassemblies

**Figure 1:**
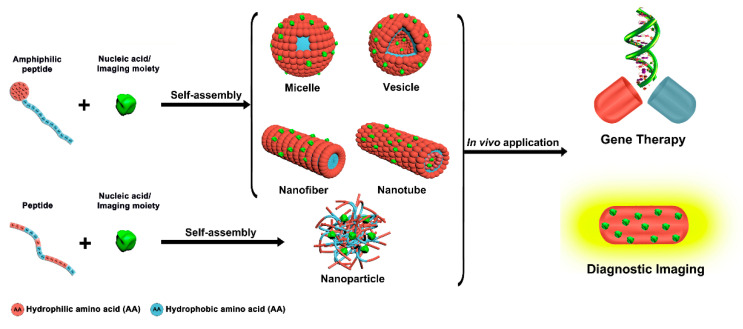
Peptide-based supramolecular nanoassemblies in gene therapy and diagnosis.

Peptide self-assembly has emerged as a promising research field in nanomedicines, due to the many advantages associated with multifunctional well-defined peptide-based nanoassemblies [21,22,23,24,25]. Common amphiphilic peptides and peptide-conjugates yielding supramolecular assemblies for biomedical applications include linear, ionic complementary, long-chain alkylated, and lipo-peptides [26]. Compared to individual peptides, peptide self-assemblies offer several unique features, like multivalent binding, dynamic control of cargo interactions, responsiveness to environmental and cellular cues, and long circulation times [27]. Various peptide-based supramolecular architectures like micelles, vesicles, nanofibers, nanotubes, and nanoparticles can be generated via a rational design of the molecular building blocks (Figure 1) [28]. As polar and nonpolar regions coexist in amphiphilic peptides, self-assembly can be readily accomplished through microphase separation [29,30].

The main driving force for the self-assembly are non-covalent interactions like hydrophobic and ionic interactions, van der Waals forces, hydrogen bonds, and π-π stacking [29]. For example, π-π stacking contributes to the self-assembly of peptides mainly via aromatic residues, whereas hydrogen bonding has a key role in the formation and stabilization of secondary structures [8]. Lateral connections between peptides with stable β-strand conformations induce extended β-sheet structures that were recognized as a main factor for the formation of nanofibers and nanotubes. In the β-sheets, the peptides are ordered such that the hydrophobic side-chains point in one direction and the polar side-chains in the other [31]. On the contrary, an α-helical structure prompts the formation of micelles and vesicles. α-helices result from hydrogen bonding between the carbonyl group of one amino acid and the amino group 4 residues further down the peptide. The side chains of the amino acids stick outward from the α-helix, where they are free to interact [28].

Accordingly, well-defined peptide nanoassemblies for gene delivery consist of three basic components, (i) hydrophobic amino acids governing the self-assembly process through intermolecular noncovalent interactions and the subsequent formation of secondary structures, and, (ii) hydrophilic amino acid residues to stabilize the specific structure in a biological environment, and (iii) positively charged amino acid residues to interact electrostatically with negatively charged nucleic acids [27]. In contrast to controlled self-assembly, peptide nanoparticles called peptiplexes spontaneously form by means of electrostatic interactions between positively charged peptide residues (lysine, arginine, and histidine) and negatively charged nucleic acids [32].

Alternatively, the chemical structure of the peptides are engineered such that the resulting nanoassemblies are specifically tailored to diagnostic imaging [33]. The two most essential features are the targeting property of the peptide and the functionalization with a detectable moiety [34].

### 2.1. Micelles

Peptide-based micelles, probably the most prominent self-assembly nanostructures in biomedicine, are closed monolayers consisting of a hydrophobic inner core surrounded by a hydrophilic outer shell (Figure 1). Direct dissolution and solvent switch are the two most common methods for micelle preparation. The spontaneous formation of these well-ordered supramolecular spherical structures with narrow size distribution occurs above a critical micellar concentration (CMC) and can be governed by temperature [35,36,37].

**Figure 2:**
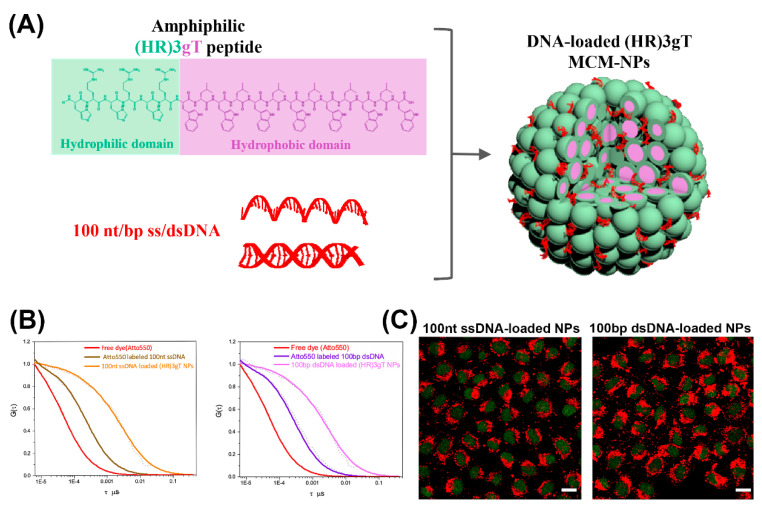
(**A**) Schematic illustration of amphiphilic (HR) 3gT peptide comprising a hydrophilic (green) and a hydrophobic (light purple) domain, and the self-assembled multi-compartment micelle nanoparticle. (**B**) Normalized fluorescence correlation spectroscopy (FCS) autocorrelation curves for fluorescently labelled 100 nt ssDNA and 100 bp dsDNA (free and loaded in multi-compartment micelles). (**C**) Confocal laser scanning microscopy (CLSM) merged images (GFP and Atto550) of H2B-GFP expressing HeLa cells treated with 100 nt/bp ss/dsDNA-loaded (HR)3gT multi-compartment micelle nanoparticles after 24 h. Adapted and modified from [40], with permission. Copyright: 2020 Royal Society of Chemistry.

For gene delivery applications, peptide-based micelles offer several advantages like high stability, high therapeutic potency due to efficient gene loading, and small size conducive to deep tumor penetration and cellular uptake [38]. Notably, formation of cationic micellar nanoassemblies increases the local positive charge density in solution, supporting an effective DNA condensation [38]. For example, cationic peptide dendrimers with side arm lipid conjugates that were able to self-assemble into micelles and condense siRNA, were introduced as a versatile platform for effective nucleic acid delivery [39]. The peptide-based micelles were non-cytotoxic at various concentrations and demonstrated superior delivery efficiency over common transfection agents, like Lipofectamine 2000 or Lipofectin^®^. In another example, a rationally designed amphiphilic peptide that self-assembled into multicompartment micelles (MCMs) was able to efficiently entrap single- and double-stranded DNA up to 100 nucleotides in length (Figure 2A,B) [40]. These purely peptidic, nanosized MCMs demonstrated rapid and efficient uptake by cells (Figure 2C).

Cationic peptide-based micelles are not only able to condense nucleic acids effectively, but they can be tailored to target specific cells and to facilitate cellular uptake, endosomal escape, and transport to the nucleus [38]. In this context, a multifunctional (CR8GPLGVH5-Pal) dipeptide comprising a cell-penetrating moiety (R8), a matrix metalloproteinase-2 (MMP-2) specific sequence (GPLGV), pH-responsive residues (H5), and a hydrophobic moiety (palmitic acid, Pal) self-assembled into micelles and condensed plasmid DNA (pDNA), resulting in an efficient vector for gene delivery [41]. Another cationic amphiphilic peptide, R3V6, that self-assembled into micelles showed remarkable potential for the combined delivery of siRNA and carmustine (an anti-cancer drug) to C6 glioblastoma cells [42]. Carmustine was efficiently entrapped into the hydrophobic core of the micelles, whereas the siRNA formed a stable complex with the positively charged amino acid residues of the hydrophilic shell.

Major efforts in the field of gene therapy were aimed at achieving enhanced stability, prolonged circulation time and controlled gene release of the nanovectors [38]. Amphiphilic cationic A12H5Kn (n = 10 or 15) peptide assembled micelles with hydrophobic alanine residues, forming the core, cationic lysine residues supporting the interaction with charged DNA and histidine residues promoting the endosomal escape of the micelles [43]. Such A12H5Kn micelles demonstrated strong DNA-binding ability and efficient protection of entrapped DNA. Consequently, HepG2 and 4T1 cell lines showed significantly higher expression of a reporter gene delivered by micelles than cells transfected with corresponding polyethilenimine/DNA complexes.

Self-assembled peptide-based micelles can also be used for disease diagnosis, in particular in cancer. Self-assembled micelles based on C-3, an amphiphilic RVRRFFF peptide coupled to a nitrobenzoxadiazole fluorophore (NBD) were developed for recognizing cancer cells with high furin expression since RVRR is a cleavage site for furin [44]. When taken up by living cells, C-3 micelles allowed the successful detection of furin by the dissociation of micelles accompanied by an increase in fluorescence. Thus, as furin was reported to be abnormally expressed in various cancers, C-3 micelles might prove valuable tools for the early diagnosis of certain cancers.

**Figure 3:**
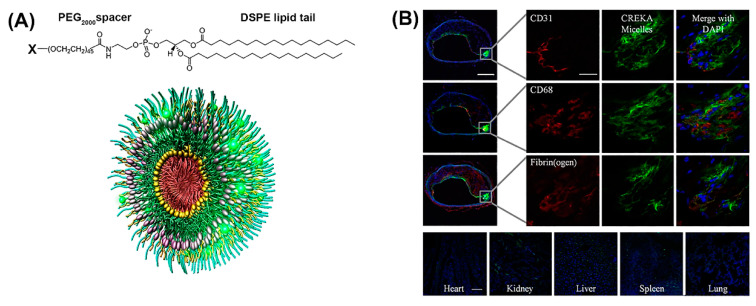
(**A**) Construction of modular multifunctional micelles. Individual lipopeptide monomers are made up of a DSPE tail, PEG_2000_ spacer, and a variable polar head group (X) of CREKA, FAM-CREKA, FAM, *N*-acetylcysteine, Cy7, or hirulog (top). The monomers were combined to form various mixed micelles. The 3D structure of FAM-CREKA/Cy7/hirulog mixed micelles (bottom). (**B**) Fluorescent microscopy images of micelle localization in the atherosclerotic plaque obtained 3 h after tail vein injection of 100 μL micelles (1 mM) (upper panels). No fluorescence was observed in the heart or lung, and only little fluorescence was observed in the kidney, spleen, and liver (scale bar, 100 µm) (bottom panels). Adapted and modified from [47], with permission. Copyright: 2009 National Academy of Sciences, USA.

Notably, peptide-based micelles can simultaneously exhibit diagnostic and therapeutic properties [45]. For example, amphiphilic peptide-based conjugates made up of a fibrin-binding peptide, CREKA, or a fluorescently labeled CREKA conjugate (Cy7-CREKA) like a cationic head group, a PEG_2000_ spacer, and a 1,2-distearoyl-sn-glycero-3-phosphoethanolamine (DSPE) hydrophobic tail, formed micelles that served for theranostic systems in cancer and atherosclerosis (Figure 3A) [46,47]. When Cy7-CREKA micelles were injected intravenously into an intracranial GL261 glioma mouse model (Figure 3B), the peptide-based micelles accumulated at the tumor site within 1 h, post injection [46]. By day 7, the majority of the micelles were cleared and the organs showed no signs of cytotoxicity or tissue damage, as determined by histological evaluation. The active binding of Cy-7 CREKA to fibrin within the glioblastoma blood vessels confirmed the tumor-homing property of these micelles.

Besides CREKA, tumor targeting in mice was also evaluated by conjugating the peptide LyP-1 (CGNKRTRGC) to DSPE-PEG_2000_ [48]. After in vivo administration of such micelles, a pronounced accumulation at the tumor site was observed, indicating that a tumor homing peptide could effectively deliver intact nanoparticles into the extravascular compartment in tumors. CREKA-DSPE-PEG_2000_ monomers were mixed with 1,2-distearoyl-sn-glycero-3-phosphoethanolamine-*N*-diethylenetriamine pentaacetic acid (gadolinium salt) (18:0 PE DTPA(Gd)) to form gadolinium containing a peptide-based cancer detection, by an improved contrast in the magnetic resonance imaging (MRI) [49].

Peptide-based micelles were engineered to respond to various internal or external stimuli, ultimately yielding functional “smart” nanostructures [3]. The major goals of stimulus-responsiveness in micelles tuned for gene delivery are to achieve enhanced or controlled gene release, improved cellular uptake, and control over the intracellular fate of nucleic acids [50,51]. For example, micelles assembled from a pH-responsive chimeric peptide, (Fmoc)2KH7-TAT, in the presence of pGL-3 reporter plasmid DNA (pDNA) and doxorubicin (DOX) were used to transfect 293T and HeLa cell lines [52]. Once enodcytosed by cells, the imidazole group of the histidine residues got protonated in the acidic environment of the endolysosomal compartment, resulting in swelling of the micelles. Besides facilitating endosomal escape, the loss of micelle compactness caused a cumulative fast release of DOX and pDNA, which effectively inhibited cell growth in vitro and significantly restrained tumor growth in vivo.

### 2.2. Vesicles

Vesicles are spherical, bilayer-delimited hollow assemblies made from one or more types of amphiphilic molecules (Figure 1) [28]. Hydrophilic domains are exposed to the inner and outer aqueous environments, whereas hydrophobic residues pack together between hydrophilic interfaces [53]. Accordingly, vesicles are capable of entrapping hydrophilic molecules in the interior aqueous phase and hydrophobic molecules in the hydrophobic parts of the bilayer [54]. The vesicle size can be tuned by adjusting the composition and chain length of the building blocks [3,55].

Self-assembly of pure amphiphilic oligopeptides [56] and diblock copolypeptides [57,58] resulted in vesicle formation in aqueous solution. The hydrophobicity of the peptide’s tail largely determines whether the peptides assemble into vesicles or nanotubes (see below). Furthermore, surfactant-like peptides with a hydrophilic head domain that consisted of aspartic acid residues, and a hydrophobic tail made of 4−10 glycine residues were shown to self-assemble into vesicles of about 30–50 nm in diameter [59,60].

**Figure 4:**
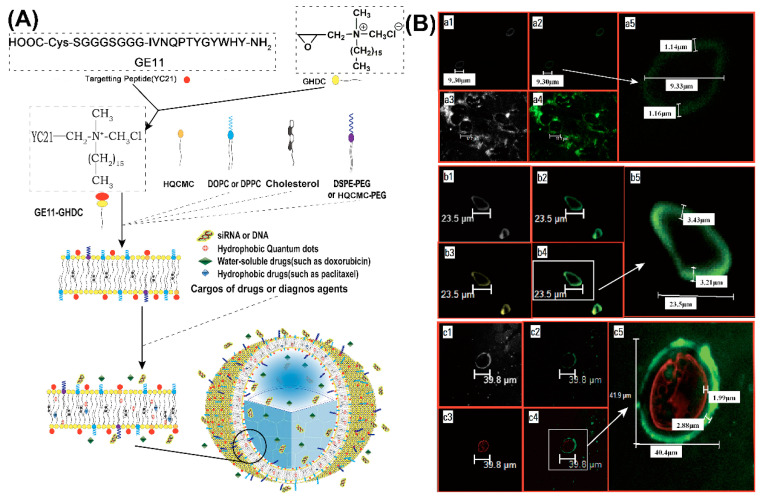
(**A**) Schematic of epidermal growth factor receptor (EGFR)-targeted self-assembled peptide vesicles (SPVs) formed from amphiphilic peptide surfactant and lipid components. (**B**) Physical and microstructural characteristics of EGFR-targeted SPVs. (**a**) Confocal images of large peptide microparticles, showing large unilamellar vesicles with an external peptide-lipid bilayer (approximately 1.15 μm thick). (**b**) Confocal images of large peptide microparticles encapsulating hydrophobic QDs (yellow fluorescence), showing large unilamellar vesicles with an external peptide-lipid bilayer (approximately 3.21 μm thick). (**c**) Confocal images of large peptide microparticles encapsulating a hydrophilic genetic payload (cy3-labelled siRNA with red fluorescence), showing large unilamellar vesicles with an external peptide-lipid bilayer (approximately 2.88 μm thick). Reproduced from [67], with permission. Copyright: 2016 Elsevier.

Due to the intrinsic chemical and biological properties of peptides, peptide-based nanovesicles offer several advantages over liposomes, such as better membrane fluidity, targeting, biocompatibility, and stability [61,62]. For in vivo delivery of DNA, peptide-mediated targeting and also the protection of the cargo against threatening extracellular factors, are of particular significance. Maintaining DNA stability and prolonging its circulation time, promote an improved organ distribution [63,64]. Self-assembled peptide vesicles (SPVs) were modified by a wide range of chemical functionalizations, including conjugating other peptides or bioactive molecules to the outer surface, or by encapsulating specific cargoes in the aqueous cavity or hydrophobic domain of the membrane [65,66]. A good representative of this kind of multifunctional SPV platform is based on the epidermal growth factor receptor (EGFR) binding peptide GE11 conjugated to glycidyl hexadecyl dimethylammonium chloride (GHDC) (Figure 4A) [67].

When cholesterol (Chol) or 1,2-dioleoyl-sn-glycero-3-phosphoethanolamine (DOPE) was added to the self-assembly of GE11-GHDC, EGFR-targeting SPVs with a stabilizing lipid bilayer structure were obtained (Figure 4B-a). Further modifications of the peptide platform were achieved by including hydrophobic (e.g., anticancer drugs, quantum dots (QD), Figure 4B-b) or water-soluble cargoes (fluorescent dyes, anticancer drugs, nucleic acids, Figure 4B-c). For example, cationic SPVs (GE11-GHDC/ HQCMC/Chol) with a high positive zeta potential were prepared for the delivery of genes or siRNAs. The inclusion of fluorescent compounds for easy localization further extends the functionalities of SPVs and turns them into theranostic platforms. GE11-GHDC-based vesicles that were functionalized, correspondingly demonstrated EGFR targeting, gene transfer, and high potency for tumor growth suppression in vivo.

Furthermore, as with micelles, peptide building blocks provide the possibility of designing vesicles that are highly sensitive to local environmental conditions (e.g., pH, redox potential, enzymes) or external stimuli (light, magnetic field, ultrasound) [57,68]. For example, cationic vesicles derived from a polypeptide with a hydrophobic poly(l-glutamate) (PLG) backbone and a pH-responsive hydrophilic poly(2-aminoethyl methacrylate hydrochloride) (PAMA) side chain loaded with Doxorubicin hydrochloride (DOX·HCl) were able to release the cargo upon a change of pH, due to protonation of PAMA side chains [69]. The interaction of cationic PLG-g-PAMA vesicles with DNA suggested that these vesicles might prove useful for the codelivery of therapeutic drugs and genes.

Poly(l-lysine hydrochloride)(PLL) and poly(gamma-benzyl-d7-l-glutamate) copolypeptides were also shown to form pH- and temperature-sensitive vesicles, when combined with pDNA [70]. In this case, the pDNA was partially condensed on the PLL phase and partially encapsulated inside the vesicle, resulting in an enhanced protection.

### 2.3. Nanofibers

Nanofibers are cylindrical structures with a length of up to microns and a width typically between 5 to 20 nm (Figure 1). The high surface-to-volume ratio provides nanofibers with a large capacity for loading various bioactive molecules, including nucleic acids [27,30]. Amyloid peptides, ionic self-complementary peptides, collagen-like triple helical peptides, and amphiphilic peptides are able to self-assemble into nanofibers [71].

Secondary structure and side-chain interactions are widely perceived to play a crucial role in the self-assembly of nanofibers. Accordingly, while considering the hydrophobic–hydrophilic balance, amino acid sequences can be tailored to promote nanofiber formation [29]. For example, nanofiber self-assembly based on short amphiphilic peptides primarily relies on highly hydrophobic amino acids (Ile or I) and their propensity for β-sheet structuring [72]. The peptide-based nanofibers (PNFs) adopt a β-sheet secondary structure to elongate into ordered nanofibers [73]. Besides sheet formation, the collapse of hydrophobic alkyl chains also promotes nanofiber assembly [74]. Accordingly, PNFs for gene delivery are composed of three main domains—(1) a hydrophobic tail, predominantly an alkyl chain, (2) a β-sheet forming peptide sequence capable of intermolecular hydrogen bonding, and (3) a hydrophilic head made out of basic amino acids that are positively charged under physiological conditions [22]. Provided the hydrophilic head is located at the exterior of the nanofibers, bioactive molecules can be entrapped within the network, during the self-assembly process [29]. Hence, PNF self-assembly and functionality profoundly depend on all three domains [75]. PNFs are promising tools for gene delivery as they can be engineered to form a platform of positively charged amino acids that electrostatically interact with nucleic acids. For example, PNFs as a non-viral vector system for siRNA delivery demonstrated effective knockdown of a targeted protein (BCL2) expression and induced apoptosis in vitro [76]. Moreover, an increased residence time as well as biological activity of siRNA was shown after administration of the PNF/siRNA complexes to the rat brain.

**Figure 5:**
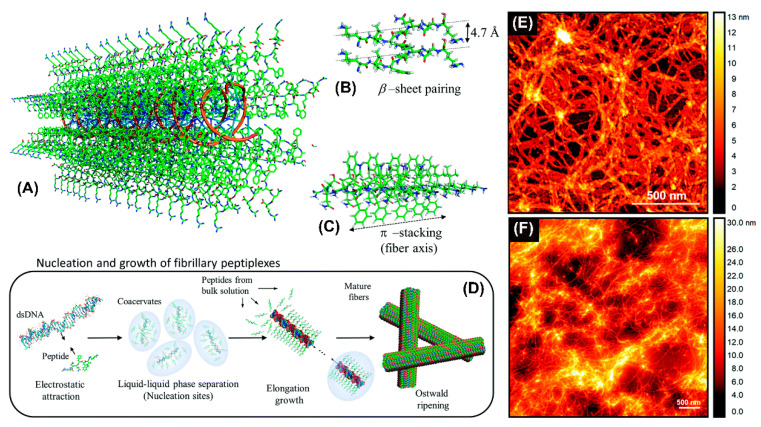
Schematic representation of the structural model proposed for complexation between KIW7 and DNA. (**A**) Fibrillary scaffold with a DNA core (orange ribbons) templating the growth of a peptide shell. (**B**) β-sheet paring between adjacent KIW7 chains in the fibers. (**C**) Supramolecular stacks formed by π-π interactions between phenylalanine and tryptophan. (**D**) Illustration of nucleation and growth of peptiplexes through liquid–liquid phase separation. AFM topography from KIW7 peptide complexed (**E**) with 200 bp DNA, and (**F**) with pDNA. Adapted and modified from [77], with permission. Copyright: 2020 Royal Society of Chemistry.

Recently, an amphiphilic peptide NH2-KIWFQNR-COOH with cationic residues at both termini to condense DNA was reported to promote the elongation of nanofibers by β-sheet pairing and β-stacking (Figure 5A–C) [77]. Fibril formation involved double-stranded DNA as a template inducing peptide fibrillization (Figure 5D). The resulting nanofibers were likely composed of DNA cores surrounded by a peptide shell. Atomic force microscopy revealed the formation of interconnected nanofiber networks for both KIW7/200bpDNA (Figure 5E) and KIW7/pDNA (Figure 5F), with nanofibers of width 20 nm. These results suggest that peptidic nanofibers through noncovalent strategies have a remarkable potential for DNA delivery.

Obviously, PNFs lend themselves to theranostic applications and have been widely employed as advanced materials for imaging [78]. Accordingly, gold nanoclusters (AuNCs) were introduced into self-assembled PNFs to enhance their luminescence efficiency. For example, in the presence of HAuCl_4_, the amphiphilic peptide (RGDAEAKAEAKCCYYCCAEAKAEAKRGD) self-assembled into a nanofiber scaffold for AuNCs where the luminescence of AuNCs was enhanced nearly 70-fold [79]. PNF-AuNCs were efficiently used for fluorescence imaging of HeLa cells, whereby the RGD recognition motif at both ends of the peptide allowed specific targeting. Similarly, fibrillar nanostructures co-assembled from different peptide amphiphiles (PA) containing a β-sheet forming hydrophobic block and a linker peptide conjugated to 1,4,7,10-tetraazacyclododecane-1,4,7,10-tetraacetic acid (DOTA) were used for improving contrast in MRI [80]. Large amounts of Gd^3+^ could be chelated to the fiber surface, suggesting that PNFs that can be potentially used as nanoprobes in magnetic resonance imaging (MRI).

### 2.4. Nanotubes

Peptide nanotubes (PNTs) are three-dimensional, highly organized systems that maintain a well-defined hollow cylindrical shape by molecular interactions of the amphiphilic building blocks (Figure 1) [81]. PNTs provide numerous possibilities for functionalization, for example at the head group of the peptide amphiphiles, and thus these versatile structures are candidates for a broad range of applications [82]. Considering that the hydrophobic alkyl chains orient towards the core and the amino acid residues to the outer surfaces, the functional groups appear on the outer surfaces of PNTs [83]. However, PNTs represent relatively a new concept in nanomedicine and therefore only few examples were reported [84].

PNT formation is mainly governed by the peptide sequence and the ensuing intermolecular hydrogen bonds between amino acid residues [83]. PNT formation from amphiphilic peptide-derived monomers naturally progresses according to well-established phase diagrams for nanotube assemblies [85]. Notably, the solution pH and hydrophobicity of peptide monomers are the prominent factors in determining PNT formation and dimensions [74,86].

Organic nanotubes based on the rational design of cyclic polypeptides were first reported by Ghadiri and coworkers [87,88]. These cyclic peptides spontaneously self-assemble into long channel structures via extension of hydrogen-bonded stacking interactions and, when exposing suitable hydrophobic residues on the surface, wrap the lipid bilayer around themselves [88]. Moreover, the transmembrane channels from self-assembling PNTs were suggested to have potential for gene delivery into living cells [88]. Similarly, cyclic cyclo-(d-Trp-Tyr) formed nanotubular assemblies in the presence of pDNA, mediating an enhanced in vitro and in vivo duodenal permeability for the pDNA [89]. These results highlighted the potential application of this oral gene delivery system for the genetic treatment of diseases associated with duodenum, stomach, liver, and kidney.

Surfactant-like peptides can also self-assemble into nanotubes with the main driving force in assembly being the formation of a polar interface that sequesters the hydrophobic tail from water contact [90]. The formation of well-defined hierarchical super-secondary structures is then induced by specific side chain hydrogen bonding interactions among β-sheets [91]. For example, a super-secondary structural template based on well-defined hydrogen bonds was developed via rational design and self-assembly of short amphiphilic Ac-I_3_XGK-NH_2_ (X = Q, S, and N) peptides [91]. The combination of hydrophobic adhesion and polar zipper formation between neighboring β-sheets rather than between β-strands within a sheet, intermeshed the β-sheets into stable, wide and flat ribbons. In another example, different surfactant-like peptides with a cationic head group (one or two Lys and His) and a hydrophobic tail of six Ala, Val, or Leu residues self-assembled when the pH values were higher than the peptide’s isoelectric point [92]. Depending on the assembly conditions and corresponding charge properties of the amino acids, nanotubes were formed. Due to their cationic nature, PNTs can bind negatively charged DNA or siRNA, making them suitable for gene delivery applications.

Furthermore, peptide–polymer or peptide-lipid hybrid nanotubes have been extensively studied as promising nontoxic nanostructures for biomedical applications [93,94]. They commonly consist of a peptidic core and a covalently attached polymeric or lipidic coating. Their biomedical applications including gene delivery were already reviewed [95].

### 2.5. Nanoparticles

The formation of stable and compact nucleic acid/peptide nanoparticles (NPs), also called peptiplexes is induced by electrostatic interactions between positively charged peptides and the negatively charged phosphate backbone of nucleic acids (Figure 1). Over the past two decades, these peptide-DNA complexes emerged as efficient gene carrier systems [96,97]. Notably, peptide-based transfection agents have several advantages over well-established polyplexes (cationic polymer-DNA complexes) or lipoplexes (cationic lipid-DNA complexes) including biocompatibility and easy synthesis process on a large scale. As compared to their lipid counterparts, peptides can be more stable with regard to oxidation, but especially benefit from countless tunability possibilities [98,99,100]. Peptiplex formation occurs when 90% negative charge of the DNA phosphate groups are neutralized by transition into the ordered phase [101]. In order to condense pDNA into NPs, a minimum of six to eight positive charges per peptide is required, although 13 or more positive charges are often needed for obtaining stable peptiplexes [101,102].

**Figure 6:**
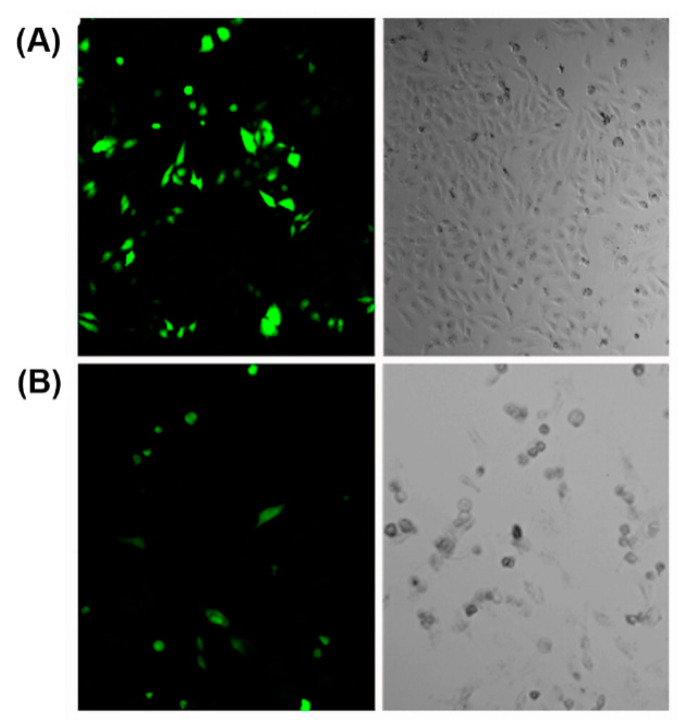
(**A**) Cells transfected with peptiplexes (N:P = 10.4) after 6 h of incubation in medium with reduced serum and 1.0 mM CaCl_2_. (**B**) Lipofectin transfected cells. Adapted from ref. [104] with permission. Copyright: 2015 American Chemical Society.

Various cationic peptides comprising different combinations of lysine, arginine and histidine residues have been exploited for nucleic acid condensation. Owing to the presence of protonable amine groups on lysine residues, lysine-rich peptides are known to effectively condense nucleic acids with a strong dependency on genetic payload concentration [103]. For example, a branched amphiphilic peptide with oligo lysine segment was able to condense GFP encoding pDNA into nanosized peptiplexes due to the strong electrostatic interactions at low peptide to pDNA ratio [104]. The compacted peptiplexes (Figure 6A), transfected HeLa cells at a higher rate than corresponding complexes with Lipofectin, a lipid-based transfection reagent (Figure 6B).

Likewise, nanoparticles built by a two-step self-assembly of globular peptide dendrimers with lysine endgroups and poly(l-leucine) carrying one glutamic acid residue, exhibited potential for gene therapy applications [105,106]. Cationic Cys-Trp-Lys_n_ peptides with 3 to 36 lysine residues were developed to systematically investigate the optimal lysine chain length for DNA condensation and transfection efficiency [107]. Peptides containing 13 or more lysines were able to strongly bind DNA and form small NPs (from 50 to 200 nm), while peptides with eight or fewer lysines formed large particles (0.7–3 µm) due to insufficient positive charges, resulting in weak DNA condensation. Interestingly, compared to Cys-Trp-Lys_19_/DNA peptiplexes, Cys-Trp-Lys_18_/DNA peptiplexes demonstrated a 1000-fold increase in transfection efficiency. However, lysine-rich peptides suffer from relatively high cytotoxicity but also from low transfection efficiency, especially if high molecular weight peptides are involved [108].

**Figure 7:**
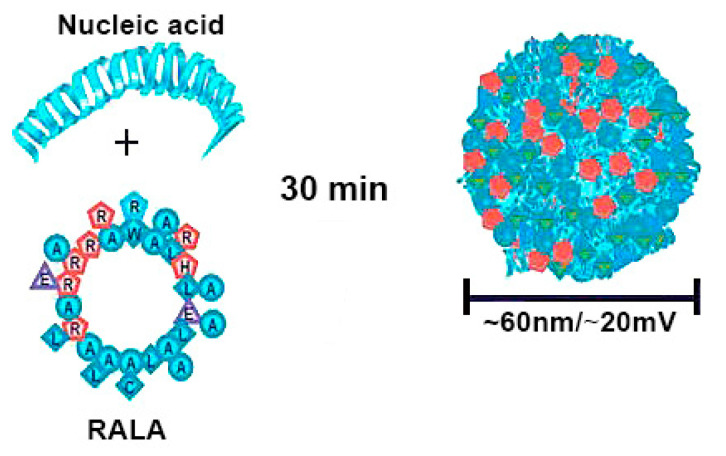
Schematic illustration of RALA/pDNA peptiplexes formation. Reproduced from [114], with permission. Copyright: 2018 Elsevier.

Arginine-rich peptides are similarly conducive to tight gene condensation, and due to their ability to promote cell penetration, they are effective delivery systems [109,110]. RALA, an amphipathic, cell penetrating peptide with seven arginines in its backbone, formed peptiplexes with siRNA as well as pDNA, which were used as delivery platform in vitro and in vivo (Figure 7) [111,112,113,114].

By the same token, hydrophilic histidine residues due to their positive charge at physiological pH, bind and condense nucleic acids in aqueous solution [115]. Moreover, protonation of its imidazole ring at low pH, functions as an effective mediator of gene delivery by promoting endosomal escape and gene release. Accordingly, short linear peptides containing multiple histidines were reported to increase DNA transfection efficiency [116], which could be further improved by using branched peptides where histidines are incorporated at higher density [117].

Interestingly, the combination of arginine and histidine was found to boost the cell penetration ability of nanoparticles and, thus, improve transfection efficacy [118]. A cationic amphiphilic K12H6V8 peptide comprising three blocks, a lysine block for DNA binding, a histidine block for endolysosomal release, a valine block as the hydrophobic component, formed peptiplexes with gene delivery properties [119]. Further modification of the cationic peptide with LHRH ligand (KHV-LHRH) resulted in significantly higher and more specific gene expression than K12H6V8 in MCF-7 cancer cells.

**Figure 8:**
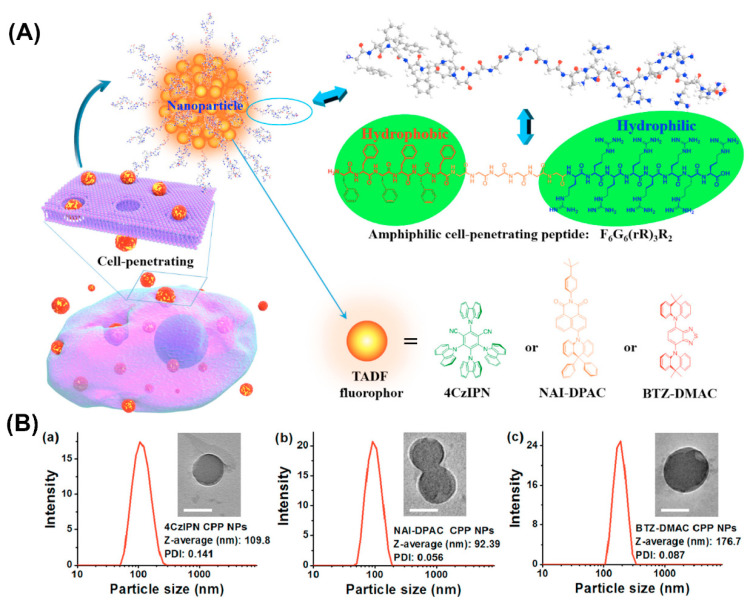
(**A**) Schematic illustration of the amphiphilic cell-penetrating F_6_G_6_(rR)_3_R_2_ peptide assembly into nanoparticles and entrapment of thermally activated delayed fluorescence (TADF) molecules. (**B**) dynamic light scattering (DLS) histograms and transmission electron microscopy (TEM) images (inset) of TADF NPs prepared at 25 μg/mL of (a) 4CzIPN, (b) NAI-DPAC, and (c) BTZ-DMAC. Scale bars = 100 nm. Reproduced from [120], with permission. Copyright: 2018 American Chemical Society.

Furthermore, amphiphilic F_6_G_6_(rR)_3_R_2_ peptide consisting of a hydrophobic amino acid sequence, a linking hexaglycine and a hydrophilic cell-penetrating sequence formed NPs that when combined with thermally activated delayed fluorescence (TADF) molecules, served for fluorescence imaging in the oxygenic environment of living cells (Figure 8) [120]. The well-dispersed NPs protected the encapsulated TADF molecules from oxidation and the cell-penetrating properties conveyed by arginine residues enabled the transport of the entrapped hydrophobic fluorophores across the cell membrane barrier.

Similarly, by using porphyrin as photoactive dye, photothermal NPs based on the self-assembly of peptide–porphyrin conjugates (TPP-G-FF) for simultaneous photo acoustic imaging and photothermal therapy were developed [121]. The FF, deduced from Alzheimer’s β-amyloid polypeptide was the core self-assembling motif driving the formation of well-defined peptide porphyrin-photothermal nanodots (PPP-NDs). Owing to strong π-stacking in the spherical PPP-NDs, the light-to-heat energy conversion was highly efficient.

## 3. In Vivo Applications of Peptide-Based Nanoassemblies for Nucleic Acid Delivery

An ideal nucleic acid delivery system should have the following general features besides application specific functionalities: (1) effectively compact and protect the genetic payload in vectors within 10–300 nm in size, (2) maintain stability from the site of administration to the site of action, (3) readily reach the target cell/tissue and enter it, and (4) in case of endocytic uptake, disrupt the endosomal membrane and release the nucleic acid such that it has access to the site of action [122,123]. What mostly hampers in vivo application of non-viral vectors is that the desired responses observed in vitro do not translate well in vivo, and that the delivery systems often prove to be toxic [33]. Peptide-based vectors offer the prospect of overcoming delivery barriers, including the host’s immune response, and can be tailored to have low cytotoxicity [15].

As described above, individual, positively charged amino acids, like lysine, histidine, and arginine or their combination in short peptide sequences can strongly influence the performance of non-viral gene vectors, due to their chemical characteristics. Thus, there are numerous peptides incorporated as functional motifs into non-viral gene delivery systems that are suited for in vivo application. However, the number of purely peptidic nanoassemblies for nucleic acid delivery in vivo is significantly lower. So far, the only pure peptidic nanoassemblies studied in vivo are peptiplexes [32]. Notably, peptiplexes intended for in vivo applications are often further modified by the covalent attachment of poly(ethylene glycol) (PEG) to reduce in vivo serum protein adsorption, increase stability of the conjugates in blood, and prolong the half-life of the genetic payload through reduced renal clearance [123,124]. With regard to nucleic acid delivery systems, addition of PEG can also have beneficial effects on nucleic acids by increasing their stability against nucleases [124,125,126].

In this section, we summarize recent developments related to the in vivo application of nanoassemblies for nucleic acid delivery that are exclusively based on peptides.

### 3.1. Peptidic Nanoassemblies with Cell-Penetrating Properties

**Figure 9:**
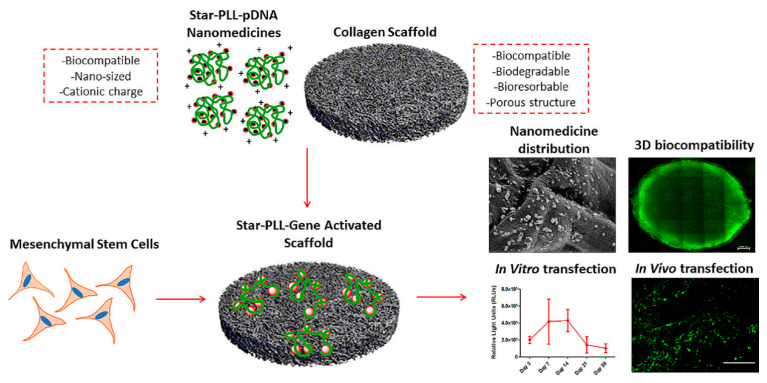
Schematic illustration and SEM micrograph of the incorporated *star*-shaped PLL/pDNA peptiplexes into collagen-based scaffold platform and in vivo transfection of autologous host cells with star-PLL/pDNA scaffold, showing prolonged, non-toxic transgene expression. Seven days post implantation, gene activated scaffolds demonstrated successful transfection of autologous host cells, as evidenced by the presence of GFP positive (green) cells. Adapted from [132], with permission. Copyright: 2019 Elsevier.

In gene therapy, the plasma membrane represents a major hurdle for the delivery of nucleic acids into the cell [14]. Cationic or amphiphilic cell penetrating peptides (CPPs) are able to mediate nucleic acid translocation through the cell membrane, without cytotoxic effects [127]. Consequently, CPPs have become a topic of interest in non-viral gene delivery and their application has dramatically increased [128,129]. Biodegradable Poly-l-lysine (PLL) derivatives were one of the first cationic cell penetrating polypeptides found to readily penetrate the cell membrane via a receptor-independent mechanism [130]. Upon interacting with nucleic acids, PLL condensed into nanoassemblies with tunable charges and molecular masses varying from a few thousand to nearly one million Daltons [32]. *An in vitro* and in vivo *comparison of* PLL/pDNA peptiplexes and SuperFect^®^ polyplexes suggested that peptiplexes have improved transfection efficacy and are a safer non-viral delivery system [131]. In another study, incorporation of *star*-shaped PLL/pDNA peptiplexes into a collagen scaffold, resulted in successful in vivo transfection of autologous host cells, *indicating the potential of this system in tissue engineering* (Figure 9) [132]. Star-shaped PLL/pDNA peptiplexes demonstrated a 2-fold increase in reporter transgene expression, compared to the widely used vector polyethylenimine, a 44-fold increase compared to a 32-armed star-PLL and a 130-fold increase compared to its linear analogue. *In addition*, PEGylated PLL minimized systemic non-specific interactions and consequently improved the delivery efficiency and safety profile of gene vectors [133]. PEGylated PLL/siRNA peptiplexes were applied as anti-angiogenesis gene therapy in hepatocellular carcinoma and showed high anti-tumor efficacy [134]. Different *PEGylated PLL/pDNA* polyplexes were introduced as a promising candidate for lung gene therapy directed at airway epithelial cells, after their administration into the airway [133].

**Figure 10:**
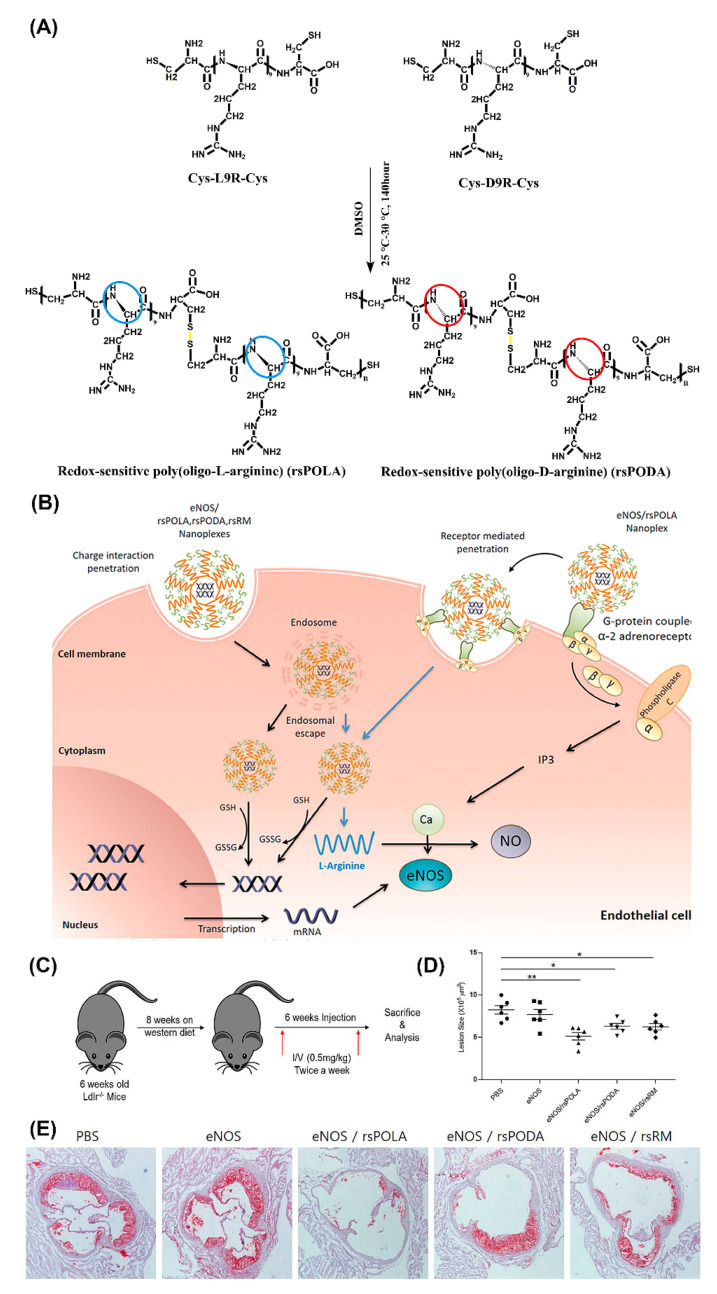
Schematic illustration of (**A**) chemical one-step synthesis of redox-sensitive poly(oligo-arginines) (rsPOLA), (**B**) targeted gene delivery of rsPOLA peptiplexes into endothelial cells, and (**C**) experimental design of evaluating peptiplexes anti-inflammatory and anti-atherogenic potency in Ldlr^−/−^ atherosclerotic mice. (**D**) Mean lesion size of sinus aorta after treatments (n = 6 animals per group). (**E**) Representative images of Oil Red O-positive atherosclerotic lesion after treatment (*n* = 6). Reproduced from [139], with permission. Copyright: 2017 Elsevier.

One of the most versatile and promising in vivo gene transfer vectors with cell penetrating properties are peptiplexes based on various lengths of oligoarginine [135]. The merits of utilizing positively charged arginine for gene delivery, with superior cell penetrating ability compared to lysine, are firmly supported by numerous studies [136,137]. For example, R15 peptiplexes delivering a β-galactosidase gene into mouse dermal tissue resulted in high levels of gene expression [138]. In another study, targeted gene-therapeutic peptiplexes comprising a redox-sensitive poly(oligo-L-arginine) (rsPOLA)/pDNA significantly reduced atherosclerotic inflammation in mice (Figure 10) [139]. The reductive environment inside the cells lead to rsPOLA disulfide bond disruption and release of the gene (Figure 10B). Lesion size measured by computer-associated morphometry revealed a 30% decrease in the mean lesion area of the aortic sinus, for groups treated with peptiplexes, compared to the control groups (Figure 10D,E). Several other in vivo studies validated reducible poly(oligo-D-arginine) (rPOA)/pDNA complexes as an efficient non-viral gene carrier in the treatment of hypoxic–ischemic brain injury, spinal cord tumors, and in ischemic heart and lung diseases [140,141,142,143].

**Figure 11:**
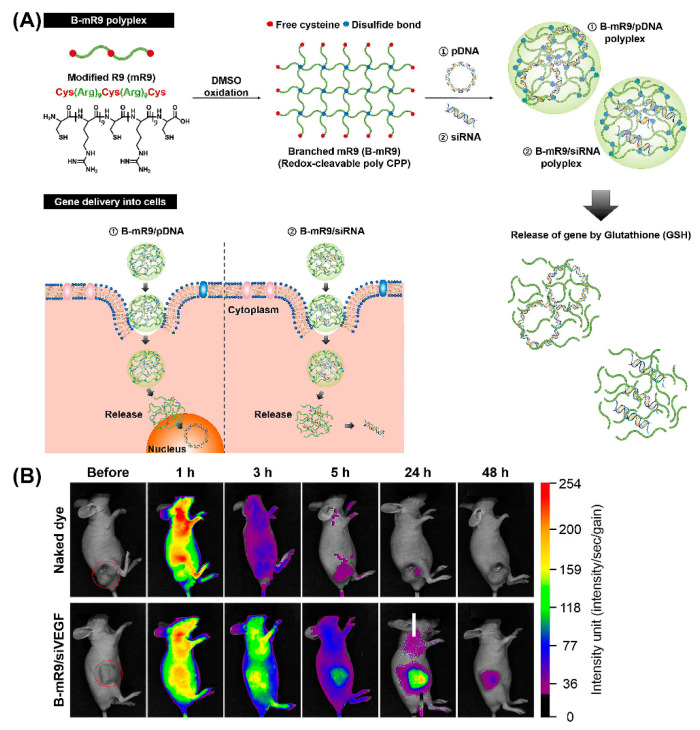
(**A**) Schematic illustration of synthesis of the branched modified R9 (B-mR9) CPP and construction of pDNA and siRNA polyplexes. Positively charged B-mR9 can be complexed with negatively charged genes through electrostatic interactions. B-mR9 polyplexes were delivered into cells by means of CPP-mediated permeabilization. The branched structures of B-mR9 were then cleaved by the reductive conditions of the cytoplasm. Finally, pDNA or siRNA was released into the nucleus or cytoplasm, respectively. (**B**) Naked dye and B-mR9/siVEGF polyplexes were intravenously injected into NCI-H460 tumor-bearing mice and the biodistribution was observed over 48 h. Adapted and modified from [148], with permission. Copyright: 2017 Elsevier.

Importantly, peptide dendrimers, highly branched and star-shaped macromolecules with great molecular uniformity and monodispersity, are well-studied examples for non-viral gene delivery in vivo, based on their cell penetrating properties, as well as their potential to facilitate intracellular delivery of the genetic payload [144,145]. For example, arginine functionalized peptide dendrimers condensing pDNA resulted in a 6-fold higher transfection efficiency than polyetherimide/DNA complexes in breast tumor models, and were well-tolerated [146]. Comparison of a series of 14 cationic peptides including linear peptides, lipopeptides, and peptide dendrimers, revealed that LTP, an arginine-rich cationic peptide dendrimer, exhibited the greatest activity in decreasing the allergic lung inflammation in mice that were injected with LTP/siRNA nanocomplexes [147]. Likewise, comparison of a linear and branched structure of a CPP comprising modified bioreducible nona-arginine (B-mR9) (Figure 11A) revealed that branched mR9/siRNA peptiplexes were superior with regard to serum stability, tumor accumulation, selective gene release and gene-silencing [148]. *Correspondingly, the in vivo* biodistribution study demonstrated that naked dye was distributed throughout the body within 1 h and was mostly cleared after 24 h while dye-labeled B-mR9/siRNA peptiplexes accumulated well at the tumor sites where they were still detectable even after 48 h (Figure 11B). Similar advantages were associated with PEG-cleavable dendrigraft PLL/siRNA polyplexes, as evidenced by a pronounced inhibition of tumor growth in vivo [149].

Interestingly, the combination of arginine and lysine residues conferred an enhanced cell penetration ability upon the peptiplexes, resulting in a higher transfection efficiency [128,136]. The in vivo gene transfer study of tissue-specific (KR)_4_ peptide derived (i.e., GM102 and GM202) peptiplexes showed a remarkable resistance of the NPs against plasma-induced disintegration and, thus, an enhanced transfection efficiency [136]. Another cell-penetrating GGG(ARKKAAKA)_4_ peptide containing arginine and lysine residues also formed complexes with pDNA or with siRNA [150]. The siRNA peptiplexes were efficiently delivered across the plasma membrane of ocular tissue in vitro and in vivo [150].

In line with the fact that that CPP complexation with pDNA results in remarkable improvement of transfection efficiency [127], intramuscular or intradermal injection of stearylated cell penetrating transportan 10 (TP10)/pDNA peptiplexes resulted in efficient gene transfection, without any associated toxicity or immunogenicity [151]. Subsequently, by introducing trifluoromethylquinoline and fatty acid modifications to TP10, a novel CPP, PepFect6 was developed to facilitate endosomal release. While PF6/luc-siRNA nanoplex-treated mice displayed reduced expression of targeted luciferase for 2 weeks (reaching 75% silencing at day 5), naked luc-siRNA was unable to induce an RNAi response. PF6/siRNA nanoplexes promoted strong siRNA-mediated gene knockdown in liver, after tail-vein and hydrodynamic injection [152].

The amphipathic cell-penetrating RICK peptide, a retro inverso form of CADY-K, formed complexes with siRNAs that entered cells via direct translocation [153]. In vivo evaluation of a PEGylated formulation of RICK/siRNA targeting cell cycle proteins demonstrated highest knock-down efficiency, highlighting its potential in cancer gene therapy.

**Figure 12:**
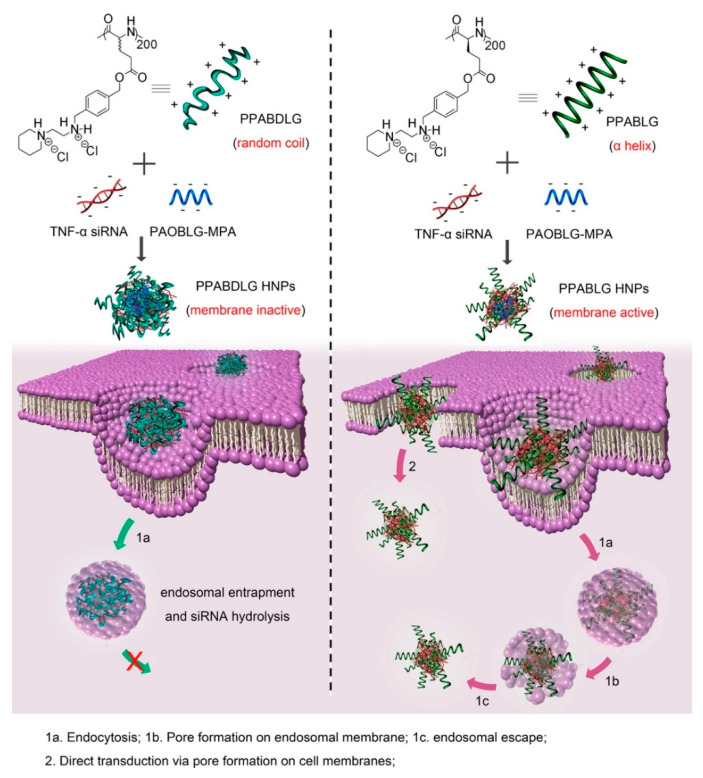
Schematic illustration of intracellular kinetics of PPABLG/PAOBLG-MPA/siRNA and PPABDLG/PAOBLG-MPA/siRNA showing PPABLG-helicity-dependent membrane disruption toward effective cellular internalization as well as endosomal escape. Adapted from [156], with permission. Copyright: 2016 American Chemical Society.

Basic amphiphilic ppTG1 and ppTG20 peptides used for pDNA delivery showed membrane-destabilizing activities that correlated with the peptides’ tendency to predominantly be in an α-helical conformation [154]. Compared to control peptides, the peptiplexes showed a higher gene transfer efficiency in the lung, 24 h after tail-vein injection. Similarly, a cationic helical cell-penetrating PVBLG-8, in combination with anionic random-coiled poly(l-glutamic acid) (PLG) polypeptides was applied for siRNA delivery [155]. PLG was incorporated in the peptide structure to promote and stabilize the polypeptide/siRNA nanocomplexes. Due to the high α-helix-dependent membrane penetration properties of PVBLG-8 and the stability of the complex, these peptiplexes overcame multiple biological barriers and revealed unique tumor targeting, tumor penetration, and selective internalization features in xenograft-bearing mice. Likewise, an essential role of helical secondary structure for plasma and endosomal membrane penetration in promoting cellular uptake and siRNA release was observed with cationic PPABLG polypeptide (Figure 12) [156]. Accordingly, systemic administration of TNF-α siRNA/PPABLG nanocomplexes lead to effective TNF-α knockdown. The remarkable anti-inflammatory properties of TNF-α siRNA/PPABLG make it a promising candidate for anti-inflammation therapies.

Furthermore, elastin-like polypeptides (ELPs) with various favorable properties such as water-solubility, biocompatibility, non-toxicity, together with reversible temperature phase transition were widely investigated for gene delivery over the past decade [157]. For instance, ELP/siRNA nanocomplexes targeting L1 cell adhesion molecules inhibited the growth of human tumor xenografts in mice [158]. Moreover, ELPs fused to a CPP for improving cellular uptake were used to deliver therapeutics into solid tumors [159]. Recently, ELPs additionally functionalized with a CPP (Tat) and IL-4 receptor targeting (AP1) moieties were used to produce ELP/siRNA peptiplexes that delivered siRNA into tumor cells in a receptor-specific fashion [160]. The gene silencing activity of these peptiplexes was confirmed in a murine breast carcinoma 4T1 allograft mouse model.

### 3.2. Peptidic Nanoassemblies Facilitating Endosomal Escape

The major uptake mechanism for various types of non-viral gene delivery systems is via endocytic pathways [161]. Therefore, endosomal escape is a crucial step in producing successful and efficient nucleic acid delivery systems [162].

**Figure 13:**
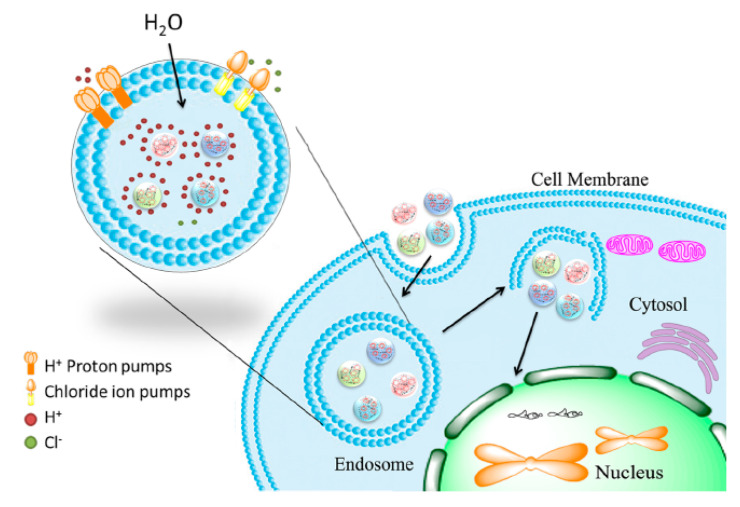
Schematic illustration of endosomal escape promoted by histidine-enriched multifunctional peptide gene vectors. Adapted from [163], with permission. Copyright: 2013 Royal Society of Chemistry.

Histidine as one of the effective mediators of endosomal escape is commonly included in peptide-based vectors. Acting as a sponge for protons at low pH (‘proton sponge effect’), histidines lead to rupturing of the endolysosomal compartment and thereby promote peptiplex escape (Figure 13) [163]. Accordingly, in vivo transfection studies of LAH4-L1, a peptide with high histidine content, complexed with siRNA, showed high gene silencing efficiency [164]. LAH4-L1 was able to promote endosomal escape of peptiplexes by destabilizing the endosomal membrane when its four histidine residues were positively charged at low pH.

**Figure 14:**
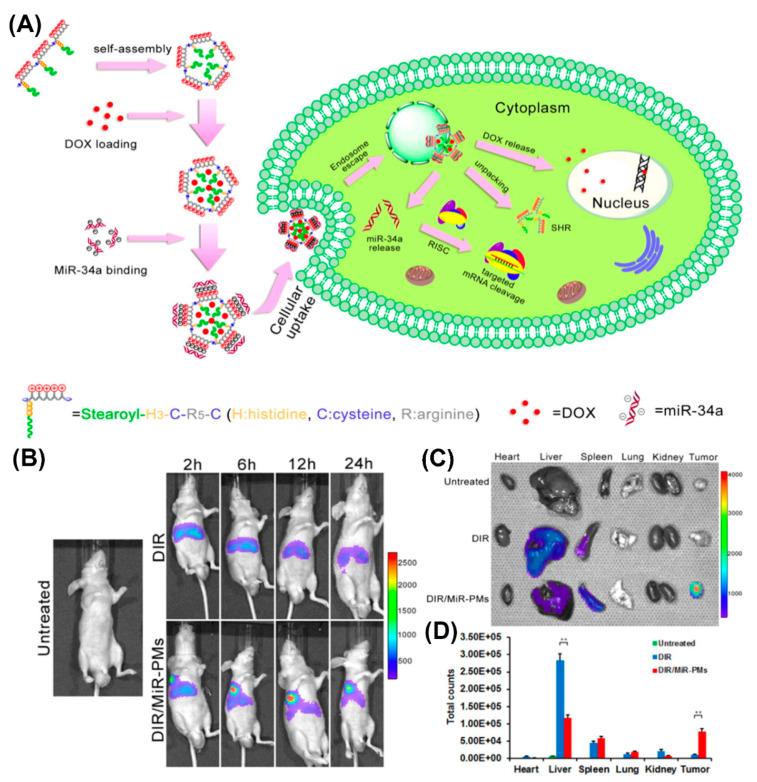
(**A**) Schematic illustration of process of doxorubicin and microRNA loading into poly(l-arginine)-poly(l-histidine)-stearoyl polyplexes and their intracellular uptake. (**B**) In vivo imaging of DU145 tumor-bearing mice after tail vein injection of DIR and DIR/MiR polyplexes at 2, 6, 12, and 24 h, respectively. (**C**) Ex vivo imaging of tumor and organs collected 24 h post-injection. (**D**) Quantification of the ex vivo tumor and organs uptake of DiR and DiR/MiR-PMs by Imaging software (Caliper Life Sciences). Reproduced from [172], with permission. Copyright: 2016 Elsevier.

With regard to in vivo applications, histidylation of various non-viral systems, including polylysine as an early example, was used to take advantage of the proton sponge effect and facilitate delivery of genetic payload to the cytosol [115,165,166,167,168,169]. Histidine improved the transfection efficiency of PEGylated PLL/siRNA nanocomplexes for silencing endogenous vascular endothelial growth factor (VEGF) expression [169]. A significantly higher tumor growth inhibition was observed in HepG2-bearing mice injected with histidylated PEG-PLL/siRNA, compared to non-histidylated counterparts. Similarly, human hypertrophic scar tissue grafts were reduced in size when treated with nanoplexes of H3K4b, an optimized peptide polymer consisting of a lysine core with four branches that contain multiple repeats of histidines and lysines, and siRNAs targeting TGF-β1 and COX-2 [170]. A corresponding HK polymer/siRNA complex was additionally conjugated to an anti-cancer drug as a novel approach for cancer therapy [171]. Likewise, reducible self-assembling poly(l-arginine)-poly(l-histidine)-stearoyl polyplexes were used for the co-delivery of microRNA and doxorubicin in androgen-independent prostate cancer treatment (Figure 14A) [172]. Incorporation of a non-invasive near-infrared probe, DIR, as an indicator in MiR polyplexes, allowed for the imaging of MiR biodistribution in tumor-bearing mice (Figure 14B). Moreover, investigation of the excised organs after 24 h revealed a strong fluorescence associated with the tumor of mice treated with DIR/MiR polyplexes, whereas tumors of untreated mice and of mice injected with free DIR showed no fluorescence (Figure 14C,D).

**Figure 15:**
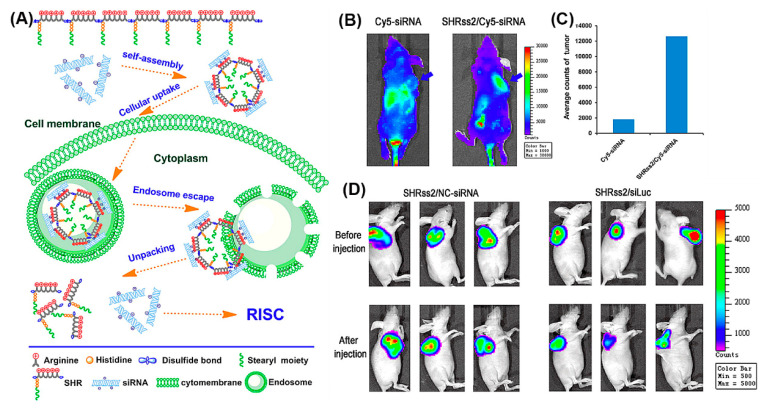
(**A**) Schematic illustration of self-assembly of cationic disulfide cross-linked SHRss and its complexation with negatively charged siRNA. (**B**) In vivo distribution and gene silencing efficiency of naked Cy5-siRNA and SHRss2/Cy5-siRNA nanocomplexes, after tail vein injection into xenograft tumor bearing mice after 4 h (blue arrows indicate the location of xenograft tumors). (**C**) Luciferase activity of the tumor tissue lysate after Cy5-siRNA and SHRss2/Cy5-siRNA nanocomplexes injections. (**D**) Luminescent images of tumors before and after SHRss2/NC-siRNA or SHRss2/siLuc nanocomplex injections. Reproduced from [173], with permission. Copyright: 2015 American Chemical Society.

The beneficial properties of arginine, histidine, and stearyl with regard to gene delivery, i.e., the propensity for membrane penetration, for promoting cellular uptake and endosomal escape, were combined in SHRss peptides (Figure 15A) [173]. SHRss2/Cy5-siRNA nanocomplexes showed 5.9 times higher mean fluorescence intensity in tumor tissue compared to free Cy5-siRNA, after their respective intravenous administration (Figure 15B,C). The in vivo luciferase activity was significantly reduced in tumor xenografts after tail vein injections of SHRss2/Luc-siRNA nanocomplexes, supporting the nanocomplexes’ ability for gene silencing (Figure 15D).

**Figure 16:**
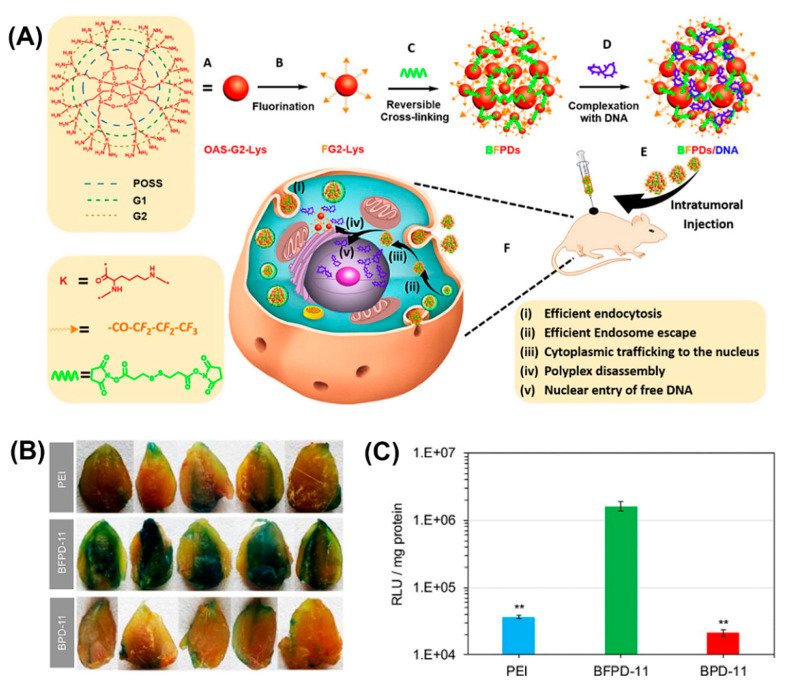
(**A**) Schematic illustrating chemical structure and preparation method of BFPDs peptiplexes for intratumoral gene delivery and their transport across the different intracellular barriers. Transfection efficacy of (**B**) pCMV*-*β-gal and (**C**) pSC-Luc, in mouse muscles after intramuscular injection of PEI, BFPD-11, and BPD-11 polyplexes for 4 days. Reproduced from [174], with permission. Copyright: 2016 American Chemical Society.

Bioreducible fluorinated peptide dendrimer (BFPDs)/pDNA polyplexes (Figure 16A) showed a superior biocompatibility in vivo and a 77-fold higher gene transfection efficiency, compared to polyetherimide/pDNA complexes (Figure 16B,C) [174].

## 4. In Vivo Applications of Peptide-Based Nanoassemblies as Probes for Diagnostic Imaging

Rapid and accurate disease diagnosis is of crucial importance for effective therapy and the prevention of disease progression to incurable stages. Current diagnostic modalities routinely used in clinical and preclinical studies include computed tomography (CT) [175], magnetic resonance imaging (MRI) [176], positron emission tomography (PET) [177], ultrasound (US) [178], optical imaging [179], and photothermal imaging [180]. The choice of diagnostic modality depends on many factors, such as the underlying pathological condition, the tissue/organ, and the intended application. For example, optical and photoacoustic imaging are suitable for studying superficial structures, endoscopy is a powerful tool for examining the walls and internal organs of the gastrointestinal tract and digestive diseases [181], non-invasive MRI, PET, and CT can be applied for whole-body imaging [182], while ultrasound is a rapid and low-cost method of examining the body’s internal organs [183].

In addition to the advantage of easy synthesis and extensive options for molecular design, self-assembling peptide nanostructures are expanding their applicability in diagnostic imaging through covalent conjugation or physical incorporation of various “imaging” agents [184]. However, developing sensitive and stable peptide nanomaterials for accurate analysis and sensitive detection of diseases remains a big challenge. We present here only the recent advances in the development of peptide-based nanoassemblies and their functionalities for in vivo diagnostic imaging, and refer to their potential in clinical applications.

### 4.1. Micelle Nanoprobes

While peptide amphiphile micelles are currently being developed for a wide range of diagnostic applications, their rapid in vivo degradation through proteolysis limit their clinical applications [185]. In this context, co-assembly of peptide amphiphiles and polymers into micelles is an effective way to enhance stability, as was shown for gadolinium-decorated pluronic F127 and peptide amphiphile (pal-AAAAHHHD) hybrid micelles [186]. Gadolinium(III) chelated by the poly(ethylene-oxide) (PEO)/peptide shell of the hybrid micelles circulated much longer than clinically established Gd-DTPA. These findings strongly back the concept of tailoring hybrid micelles to specific requirements, through simple variation of their composition rather than through synthesis of new materials.

**Figure 17:**
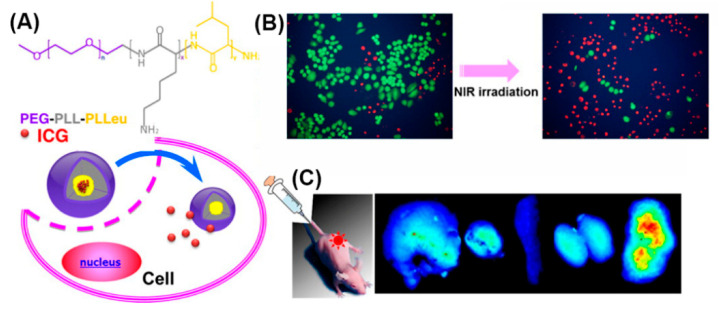
(**A**) Chemical structure of the PEG-PLL-PLLeu copolymer, formation of the micelle and the release behavior of ICG in cells. (**B**) Survival of H460 cells without and with 808 nm laser irradiation (green, calcein AM; red, propidium iodide). (**C**) Fluorescence images of organs and tumors in NCI-H460 tumor-bearing mice after the injection of ICG-labeled PEG-PLL-PLLeu micelles. Adapted from [187], with permission. Copyright: 2013 American Chemical Society.

In addition, as PEGylation improves water solubility, stability, and circulation time [125], micelles self-assembled from amphiphilic PEG-polypeptide hybrid triblock copolymers (PEG-PLL-PLLeu) that contained a near-infrared (NIR) fluorescence dye (indocyanine green; ICG) were used for NIR fluorescence imaging (Figure 17) [187]. Optical imaging using NIR light represents a new imaging modality allowing for visualizing deep-tissue structures. In vivo experiments demonstrated exact tumor localization and prolonged circulation of the NIR micelles.

### 4.2. Nanofibers Improve the Accumulation of Contrast Agents

The high surface-area to volume ratio of peptide-based nanofibers that allows for the extensive encapsulation of imaging agents, makes them attractive for diagnostic applications. In addition, nanofibers based on their size and geometry are prone to being retained in complex tissue environments.

**Figure 18:**
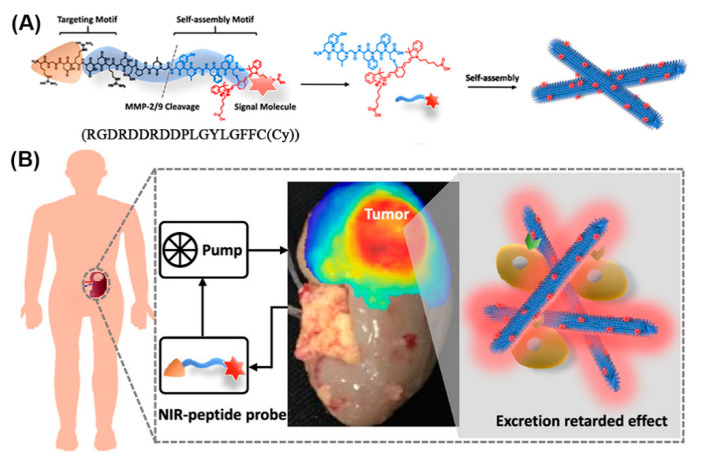
(**A**) Modular molecular design of the NIR peptide and the self-assembly of the peptide after cleavage by MMP-2/9. (**B**) NIR fluorescence image of tumor-bearing human kidney tissue after ex vivo perfusion for 2 h. Adapted and modified from [188], with permission. Copyright: 2020 American Chemical Society.

Given appropriate conditions, a peptide comprising an RGD recognition motif for targeting integrins (which are highly expressed in renal cell carcinoma (RCC)), a cleavage site for matrix metalloproteinases (MMP)-2/9 (which are typically overexpressed in the tumor microenvironment), and an NIR moiety (Cy) for signaling, self-assembled to nanofibers in solution (Figure 18) [188]. When injected into mice bearing orthotopic RCC xenografts, the RGD targeted the peptide monomers to the tumor where they were cleaved by MMP-2/9 and gradually self-assembled into nanofibers in situ. Owing to their size and interactions, the nanofiber probes were retained in the tumor microenvironment, enabling high-performance imaging of the RCC. In addition, lesions below 1 mm were identified in an ex vivo human tumor-bearing kidney, emphasizing the potential of nanofibers in clinical image-guided surgery.

Another NIR dye-labeled peptide (P18-PLGVRGRGD) forming nanofibers at tumor sites was used in photoacoustic imaging (PA) [181,189,190,191]. This peptide comprised an RGD for tumor targeting and a sequence (PLGVRG) that was cleaved by gelatinase overexpressed in the tumor microenvironment. Subsequently, fibrous nanostructures self-assembled, which exhibited an enhanced PA signal and a prolonged retention time in tumors.

A study involving a mitochondria targeting tripeptide, Mito-FF, and its mirror pair Mito-ff revealed that either enantiomer self-assembled narrow fibers of 10 nm width, while heterochiral assembly (Mito-FF/ff) produced superfibrils of around 100 nm in diameter, and 0.5–1 μm in length, due to strong backbone interactions between opposite enantiomers and strong interfiber interactions. Superfibril formation through co-assembly of racemic mixtures (Mito-Rac) was observed inside mitochondria, resulting in disrupted mitochondria both in vitro and in tumor xenografts [192]. In addition, SPECT/CT (single-photon emission computed tomography combined with computed tomography) imaging revealed an efficient tumor localization of peritumorally applied ^123^I-radiolabeled Mito-FY and Mito-fy peptides. This study unveiled the potential of a chirality in tuning supramolecular peptide assembly for biological applications.

**Figure 19:**
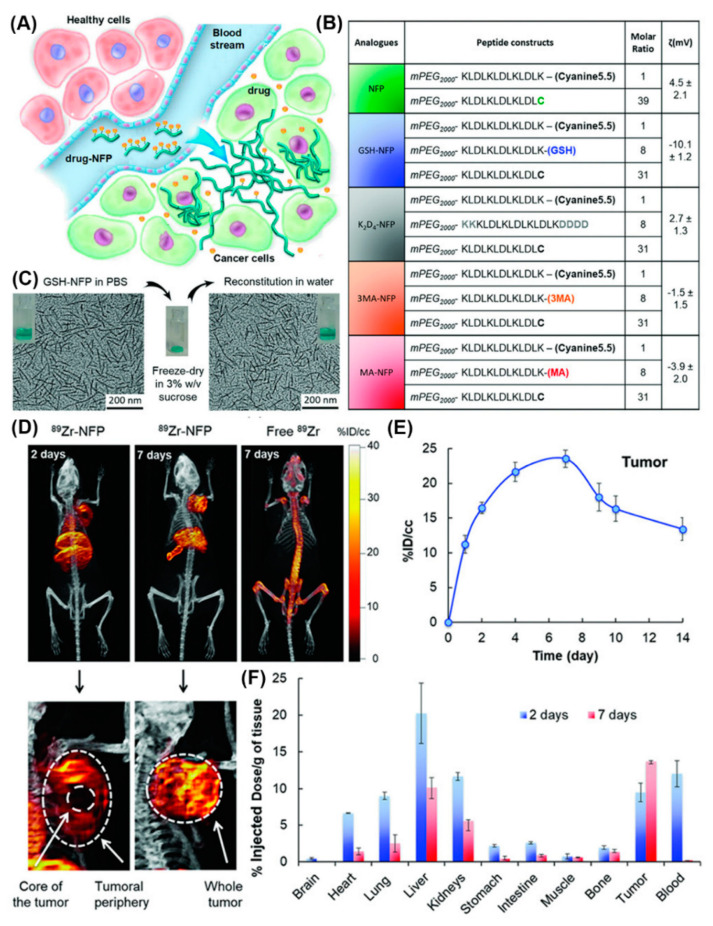
(**A**) Design of NFP analogues to improve tumoral uptake, penetration, and local retention. (**B**) A table showing NFP analogue composition and surface charge. (**C**) Morphologies of GSH-NFP in PBS buffer (left) before freeze-drying in sucrose and after reconstitution in water (right). (**D**) Representative PET/CT whole body images of SCID mice bearing MDA-MB-468 tumors were acquired 2 and 7 days after injection. (**E**) Quantification of nanofibers (radioactivity) at the region of interest (ROI; tumor) was plotted to reveal the kinetic uptake and the retention properties of GSH-NFP at tumor sites. (**F**) The biodistribution of ^89^Zr-NFP was determined in separate groups of animals. Reproduced from [78], with permission. Copyright: 2018 John Wiley & Sons.

Furthermore, a series of nanofibers based on self-assembling peptide analogues was tested for tumoral uptake and biodistribution in vivo, in real-time, through PET/CT imaging (Figure 19) [78]. All peptide precursors were PEGylated to reduce nanofiber removal through the reticuloendothelial system and had the ability to structurally evolve into an interfibril network for prolonging the retention on site. PET/CT imaging employing ^89^Zr-labeled GSH-NFP, the analogue with the most effective features in terms of tumoral delivery and drug retention, showed that the nanofibers accumulated at the tumor periphery and in the main organs, and were cleared after 7 days. Notably, based on the multifunctionality of the nanofibers they did not require a targeting ligand to achieve tumor-specific biodistribution, which expands their applications to cancers that lack cognate receptors.

Peptides that change conformation in response to external stimuli are of particular interest in diagnosis. For example, peptides that respond to the acidic pH typical for tumor microenvironments (pH 5.5–6.0) might prove useful in cancer detection. For example, a pH-responsive peptide, BP-FFVLK-PEG-His6, which at pH 7.4 self-assembled nanoparticles and transformed to form nanofibers at pH 6.5, was used for the fluorescence imaging [193]. When the nanoparticles were injected into tumor-bearing mice, the acidic tumor microenvironment triggered a change of hydrophobic/hydrophilic balance in BP-FFVLK-PEG-His6, leading to the formation of nest-like nanofibrillar clusters that covered the tumor region and had retention times up to 96 h. Similarly, pH-trigged self-assembled peptide conjugates consisting of a hydrophilic peptide (STP: SKDEEWHKNNFPLSP) and a hydrophobic tetraphenylethylene molecule, were designed for fluorescence imaging [194]. The morphology of the self-assembled conjugates that changed from nanoparticles, resulted in the accumulation of more signal molecules at the tumor site. Similarly, the transformation of Ppdf-Gd-based nanoparticles to nanotubes triggered by MMP-2-cleavage, effectively improved the accumulation and retention of a GD-based contrast agent (Gd-DOTA) in the tumor [195].

### 4.3. Nanotubes Offer More Opportunity for Functionalization

Peptide-based nanotubes are promising materials for diagnosis because their surface properties including charge and hydrophobicity/hydrophilicity can be easily modulated, expanding their versatility for encapsulating imaging agents or other bioactive compounds [95]. A prominent family of nanotubes are self-assembling cyclic peptide nanotubes (CPNTs). Due to the cyclic nature of the peptide building blocks, nanotubes offer several opportunities for functionalization. Multiple modifications on the periphery [196,197] and also on the interior of CPNTs were reported [198]. More recently, pH and redox responsive CPNTs were developed [199]. Nanotubes obtained through π-stacking of a cyclic peptide (cyclo[Gln-(d-Leu-Trp)4-d-Leu], CP) formed artificial channels in membranes, for delivery of anticancer drugs in liposomes and cancer cells [200]. Even though CPs are known for their antibacterial, antiviral, and anticancer effects in vitro, in vivo applications are rarely investigated, probably because CPs non-selectively bind to hydrophobic domains, i.e., target all cell membranes and, thus, are toxic. In vivo imaging using ^125^I-labelled CP directly injected into tumors revealed that the radioactivity was localized to the tumor tissues, with negligible translocation to other tissues. However, no direct evidence of nanotubes associated with the cancer cells or channel-mediated entry of compounds, was provided. For in vivo applications, CPNT development still met many challenges, including reducing their toxicity and improving specificity.

### 4.4. Nanoparticles for Improved Signaling

**Figure 20:**
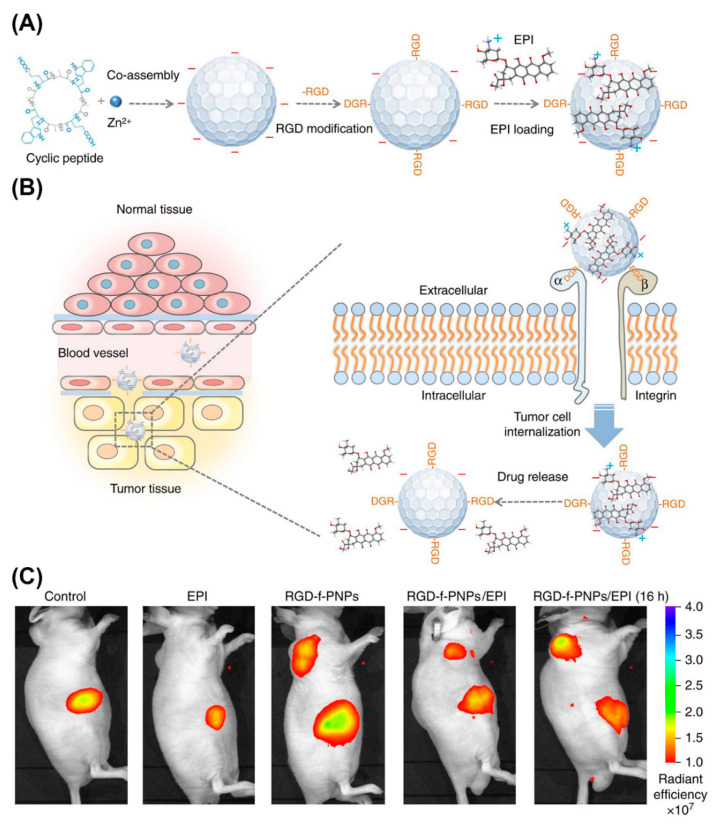
Schematic illustration of the synthesis of RGD-f-PNPs/EPI and its targeted EPI delivery into EC cells. (**A**) The RGD-f-PNPs were first synthesized by co-assembly Zn2+ ions and cyclo[-(d-Ala-l-Glu-d-Ala-l-Trp)2-] peptides, and then modified with RGD peptide moieties onto the surface of f-PNPs. The EPI was loaded with RGD-f-PNPs through π-π stacking and electrostatic interactions. (**B**) The EPI-loaded RGD-f-PNPs can be used for targeted imaging and destruction of EC cells due to their capability of active targeting and enhanced penetration. (**C**) NIR fluorescence imaging of EC-bearing nude mice after tail vein injection of saline, EPI alone, RGD-f-PNPs, and RGD-f-PNPs/EPI. Reproduced from [201], with permission. Copyright: 2018 Spring Nature.

Peptide NPs are rapidly taking root as nanoprobes for in vivo imaging. For example, NIR fluorescent peptide nanoparticles (RGD-f-PNPs) were suited for theranostic applications (Figure 20) [201]. These NPs formed by co-assembly of cyclic octa-peptide cyclo-[(d-Ala-l-Glu-D-Ala-l-Trp)2-] with Zn^2+^ ions and signaling is provided by the intrinsic optical properties of the peptide rather than by integrating fluorophores or quantum dots. Targeting to esophageal cancer (EC) cells and tumor tissues in corresponding nude mice was achieved by post-assembly conjugating RGD to the NPs. In addition, RGD-f-PNPs were loaded with epi-drugs through π-π stacking and electrostatic interactions. Drug delivery to tumor sites and the therapeutic responses could be monitored in tumor-bearing nude mice injected with RGD-f-PNPs/EPI through near-infrared fluorescence imaging.

**Figure 21:**
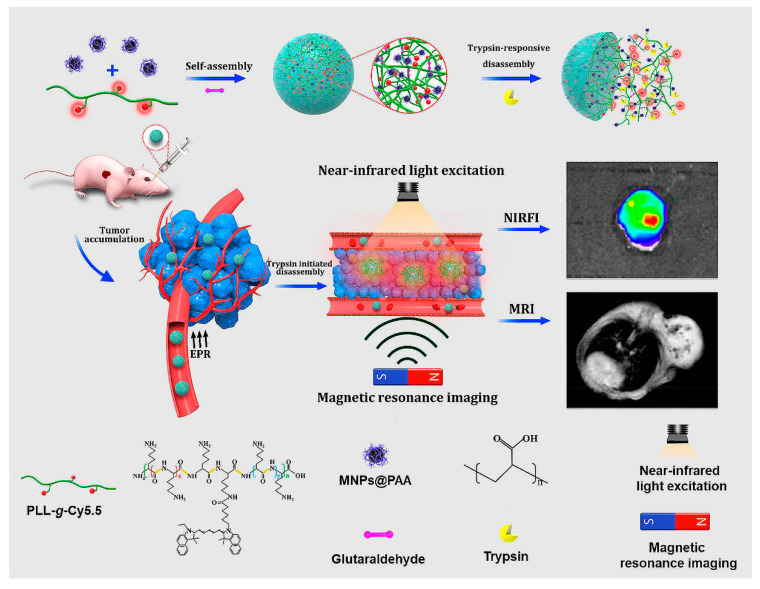
Self-assembly and trypsin-initiated disintegration of the nanoparticles for trypsin mapping of malignant tumors via NIR fluorescence and MR dual imaging. Adapted from [202], with permission. Copyright: 2020 American Chemical Society.

Self-assembly of cy5.5-modifed poly-_L_-lysine (PLLs-g-Cy5.5) and PAA decorated Fe_3_O_4_ magnetic nanoparticles (MNPs@PAA) through electrostatic interactions, in the presence of crosslinker, produced NPs for NIR fluorescence and magnetic resonance imaging (MRI) (Figure 21) [202]. The trypsin-responsive nanoparticles selectively disintegrated upon hydrolysis of PLL by trypsin, resulting in an 18-fold amplification of the NIR fluorescence and the enhancement of the MRI signal. In vivo studies showed that the probes could be used to map malignant tumors overexpressing trypsin via NIR fluorescence and MR dual imaging.

Enzyme activation (i.e., dephosphorylation), albeit of the fluorogenic moiety, was also exploited in another small molecule NIR Fluorescence/MR imaging probe (P-CyFF-Gd) [203]. In situ induced self-assembly of NPs led to an enhanced NIR fluorescence and MRI by simultaneously increasing the local density of CyFF-Gd and prolonging the retention of the signaling entity. Alkaline phosphatase triggered fluorogenic reaction and self-assembly of CyFF-Gd into nanoparticles in mice, enabled real-time, high-sensitivity imaging, and localization of the ALP activity at high spatial-resolution.

Recent advances in nanotechnology led to the development of different types of self-assembled peptide-based nanomaterials for diagnostic applications. However, the majority of studies still stay at the cellular level, and the in vivo application of peptide-based nanoassemblies remains challenging. Above, we presented some in vivo examples that provide cues on peptide designs and co-assembly with other modules that hold the key to successful imaging. More efforts should focus on the development of multimodality to enhance the in vivo imaging sensitivity and accuracy. Although peptide-based nanoassemblies still have a way to go for clinical diagnosis, recent developments in preclinical studies have definitely moved them closer to becoming a reality in the near future.

## 5. Conclusions

We presented peptide-based nanomaterials (PBNs) and their uses mainly for diagnostic imaging and gene delivery purposes. We focused on peptides because they have properties that make them particularly suited for these applications: (1) as major structural elements in all living systems, they are inherently biocompatible, which is vital for in vivo applications, (2) they can take on multiple bio-functional roles via different compositions of residues or ensuing conformations, side chain modifications, and conjugates that can be exploited for developing versatile multifunctional nanoassemblies, and (3) can serve as recognition and targeting moieties to mediate binding to specific disease-related markers, which is crucial for diagnostic imaging as well as for targeted gene therapy.

With regard to clinical applications of PBNs, we are aware of only a few that were successfully used for diagnosis in humans [204], while peptide-mediated gene delivery systems are still in their infancy. For example, ^124^I-cRGDY-PEG-C dots were used as a probe in hybrid PET-optical imaging for lesion detection, cancer staging, and treatment [205]. Evidently, the lack of advanced peptide-mediated gene delivery systems in clinics highlight the urgency of advancing this class of nanostructures.

Considering that not only peptidic but also lipidic-, polymeric, or inorganic-components are incorporated in the majority of PBNs structures, many issues concerning toxicity and thermodynamic stability can be attributed to the non-peptidic part. Therefore, tailoring PBNs for specific clinical applications remains a major challenge. One possible solution to sidestep concerns related to non-peptidic components is to develop nanomaterials exclusively based on peptides.

Several parameters should be taken into account when developing future nanoformulations for safe and effective imaging and targeted gene delivery, or even the combination thereof. First and foremost, PBNs should be target-specific, sensitive, and should have long-lasting in vivo stability, while avoiding side effects. For successful gene delivery, PBNs should be efficiently internalized and then overcome various intracellular barriers, such as the nuclear membrane in the case of gene delivery. Thus, a better understanding of the fate of PBNs inside cells and their interaction with intracellular transport mechanisms is required.

Synthesis of such advanced PBNs would require multiple steps in the laboratory, which ultimately result in high manufacturing costs. Molecular self-assembly, on the other hand, allows for developing a variety of thermodynamically stable, defined shapes, and functions, at a quicker rate and a cheaper cost than chemical synthesis. Combining the advantages of self-assembly with the beneficial properties of peptides is expected to pave the way for future human clinical applications of self-assembled PBNs in diagnosis and gene therapy.

## References

[1] Katyal P., Meleties M., Montclare J.K. (2019). Self-Assembled Protein- and Peptide-Based Nanomaterials. ACS Biomater. Sci. Eng..

[2] Qi G.-B., Gao Y.-J., Wang L., Wang H. (2018). Self-Assembled Peptide-Based Nanomaterials for Biomedical Imaging and Therapy. Adv. Mater..

[3] Habibi N., Kamaly N., Memic A., Shafiee H. (2016). Self-assembled peptide-based nanostructures: Smart nanomaterials toward targeted drug delivery. Nano Today.

[4] Chen J., Zou X. (2019). Self-assemble peptide biomaterials and their biomedical applications. Bioact. Mater..

[5] Saccardo P., Villaverde A., González-Montalbán N. (2009). Peptide-mediated DNA condensation for non-viral gene therapy. Biotechnol. Adv..

[6] Hagel L., Jagschies G., Sofer G., Hagel L., Jagschies G., Sofer G. (2008). 9-Basic Properties of Peptides, Proteins, Nucleic Acids and Virus Particles. Handbook of Process Chromatography (Second Edition).

[7] Chuah J.-A., Matsugami A., Hayashi F., Numata K. (2016). Self-Assembled Peptide-Based System for Mitochondrial-Targeted Gene Delivery: Functional and Structural Insights. Biomacromolecules.

[8] Wang J., Liu K., Xing R., Yan X. (2016). Peptide self-assembly: Thermodynamics and kinetics. Chem. Soc. Rev..

[9] Yu C.-Y., Huang W., Li Z.-P., Lei X.-Y., He D.-X., Sun L. (2016). Progress in Self-assembling Peptide-based Nanomaterials for Biomedical Applications. Curr. Top. Med. Chem..

[10] Ulijn R.V., Smith A.M. (2008). Designing peptide based nanomaterials. Chem. Soc. Rev..

[11] Alipour M., Hosseinkhani S., Weissig V., Elbayoumi T. (2019). Design, Preparation, and Characterization of Peptide-Based Nanocarrier for Gene Delivery. Pharmaceutical Nanotechnology: Basic Protocols.

[12] Riley M.K., Vermerris W. (2017). Recent Advances in Nanomaterials for Gene Delivery—A Review. Nanomaterials.

[13] Kumar S.R., Markusic D.M., Biswas M., High K.A., Herzog R.W. (2016). Clinical development of gene therapy: Results and lessons from recent successes. Mol. Ther. Methods Clin. Dev..

[14] Kang Z., Meng Q., Liu K. (2019). Peptide-based gene delivery vectors. J. Mater. Chem. B.

[15] Shahbazi-Gahrouei D., Moradi Khaniabadi P., Moradi Khaniabadi B., Shahbazi-Gahrouei S. (2019). Medical imaging modalities using nanoprobes for cancer diagnosis: A literature review on recent findings. J. Res. Med. Sci. Off. J. Isfahan Univ. Med. Sci..

[16] Mazumder S., Pavurala N. (2016). A Review on Nanoprobes for Sensing, Imaging and Disease Detection. J. Mater. Sci. Nanotechnol..

[17] Jarzyna P.A., Gianella A., Skajaa T., Knudsen G., Deddens L.H., Cormode D.P., Fayad Z.A., Mulder W.J.M. (2010). Multifunctional imaging nanoprobes. Wiley Interdiscip. Rev. Nanomed. Nanobiotechnol..

[18] Cai W., Chen X. (2007). Nanoplatforms for Targeted Molecular Imaging in Living Subjects. Small.

[19] Zhang P., Cui Y., Anderson C.F., Zhang C., Li Y., Wang R., Cui H. (2018). Peptide-based nanoprobes for molecular imaging and disease diagnostics. Chem. Soc. Rev..

[20] Ren C., Wang Z., Wang Q., Yang C., Liu J. (2020). Self-Assembled Peptide-Based Nanoprobes for Disease Theranostics and Disease-Related Molecular Imaging. Small Methods.

[21] Panda J.J., Chauhan V.S. (2014). Short peptide based self-assembled nanostructures: Implications in drug delivery and tissue engineering. Polym. Chem..

[22] Dehsorkhi A., Castelletto V., Hamley I.W. (2014). Self-assembling amphiphilic peptides. J. Pept. Sci..

[23] Santis E.D., Ryadnov M.G. (2015). Peptide self-assembly for nanomaterials: The old new kid on the block. Chem. Soc. Rev..

[24] Acar H., Srivastava S., Chung E.J., Schnorenberg M.R., Barrett J.C., LaBelle J.L., Tirrell M. (2017). Self-Assembling Peptide-Based Building Blocks in Medical Applications. Adv. Drug Deliv. Rev..

[25] Zhao X., Pan F., Xu H., Yaseen M., Shan H., Hauser C.A.E., Zhang S., Lu J.R. (2010). Molecular self-assembly and applications of designer peptide amphiphiles. Chem. Soc. Rev..

[26] Löwik D.W.P.M., Hest J.C.M. (2004). van Peptide based amphiphiles. Chem. Soc. Rev..

[27] Wang H., Feng Z., Xu B. (2019). Supramolecular Assemblies of Peptides or Nucleopeptides for Gene Delivery. Theranostics.

[28] Song Z., Chen X., You X., Huang K., Dhinakar A., Gu Z., Wu J. (2017). Self-assembly of peptide amphiphiles for drug delivery: The role of peptide primary and secondary structures. Biomater. Sci..

[29] Yadav S., Sharma A.K., Kumar P. (2020). Nanoscale Self-Assembly for Therapeutic Delivery. Front. Bioeng. Biotechnol..

[30] Fan T., Yu X., Shen B., Sun L. (2017). Peptide Self-Assembled Nanostructures for Drug Delivery Applications. J. Nanomater..

[31] Edwards-Gayle C.J.C., Hamley I.W. (2017). Self-assembly of bioactive peptides, peptide conjugates, and peptide mimetic materials. Org. Biomol. Chem..

[32] Tomich J.M., Wessel E., Choi J., Avila L.A., Filice M., Ruiz-Cabello J. (2019). Chapter 8-Nonviral Gene Therapy: Peptiplexes. Nucleic Acid Nanotheranostics.

[33] Wang W., Hu Z. (2019). Targeting Peptide-Based Probes for Molecular Imaging and Diagnosis. Adv. Mater..

[34] Lee S., Xie J., Chen X. (2010). Peptide-based Probes for Targeted Molecular Imaging. Biochemistry.

[35] Torchilin V.P. (2007). Micellar nanocarriers: Pharmaceutical perspectives. Pharm. Res..

[36] Tu R.S., Tirrell M. (2004). Bottom-up design of biomimetic assemblies. Adv. Drug Deliv. Rev..

[37] Sigg S.J., Postupalenko V., Duskey J.T., Palivan C.G., Meier W. (2016). Stimuli-Responsive Codelivery of Oligonucleotides and Drugs by Self-Assembled Peptide Nanoparticles. Biomacromolecules.

[38] Wang H., Ding S., Zhang Z., Wang L., You Y. (2019). Cationic micelle: A promising nanocarrier for gene delivery with high transfection efficiency. J. Gene Med..

[39] Kokil G.R., Veedu R.N., Le B.T., Ramm G.A., Parekh H.S. (2018). Self-assembling asymmetric peptide-dendrimer micelles—A platform for effective and versatile in vitro nucleic acid delivery. Sci. Rep..

[40] Tarvirdipour S., Schoenenberger C.-A., Benenson Y., Palivan C.G. (2020). A self-assembling amphiphilic peptide nanoparticle for the efficient entrapment of DNA cargoes up to 100 nucleotides in length. Soft Matter.

[41] Ryu K., Lee G.J., Choi J., Kim T., Kim T. (2015). Self-Assembling Multifunctional Peptide Dimers for Gene Delivery Systems. Adv. Mater. Sci. Eng..

[42] Yi N., Oh B., Ah Kim H., Lee M. (2013). Combined delivery of BCNU and VEGF siRNA using amphiphilic peptides for glioblastoma. J. Drug Target..

[43] Wiradharma N., Khan M., Tong Y.W., Wang S., Yang Y.-Y. (2008). Self-assembled Cationic Peptide Nanoparticles Capable of Inducing Efficient Gene Expression In Vitro. Adv. Funct. Mater..

[44] Li X., Cao C., Wei P., Xu M., Liu Z., Liu L., Zhong Y., Li R., Zhou Y., Yi T. (2019). Self-Assembly of Amphiphilic Peptides for Recognizing High Furin-Expressing Cancer Cells. ACS Appl. Mater. Interfaces.

[45] Accardo A., Tesauro D., Morelli G. (2013). Peptide-based targeting strategies for simultaneous imaging and therapy with nanovectors. Polym. J..

[46] Chung E.J., Cheng Y., Morshed R., Nord K., Han Y., Wegscheid M.L., Auffinger B., Wainwright D.A., Lesniak M.S., Tirrell M.V. (2014). Fibrin-binding, peptide amphiphile micelles for targeting glioblastoma. Biomaterials.

[47] Peters D., Kastantin M., Kotamraju V.R., Karmali P.P., Gujraty K., Tirrell M., Ruoslahti E. (2009). Targeting atherosclerosis by using modular, multifunctional micelles. Proc. Natl. Acad. Sci. USA.

[48] Karmali P.P., Kotamraju V.R., Kastantin M., Black M., Missirlis D., Tirrell M., Ruoslahti E. (2009). Targeting of albumin-embedded paclitaxel nanoparticles to tumors. Nanomed. Nanotechnol. Biol. Med..

[49] Federico Pineda E.J.C. (2014). Fibrin-Targeting, Peptide Amphiphile Micelles as Contrast Agents for Molecular MRI. J. Cell Sci. Ther..

[50] MacEwan S.R., Callahan D.J., Chilkoti A. (2010). Stimulus-responsive macromolecules and nanoparticles for cancer drug delivery. Nanomedicine.

[51] Lee D., Rejinold N.S., Jeong S.D., Kim Y.-C. (2018). Stimuli-Responsive Polypeptides for Biomedical Applications. Polymers.

[52] Han K., Chen S., Chen W.-H., Lei Q., Liu Y., Zhuo R.-X., Zhang X.-Z. (2013). Synergistic gene and drug tumor therapy using a chimeric peptide. Biomaterials.

[53] Kita-Tokarczyk K., Grumelard J., Haefele T., Meier W. (2005). Block copolymer vesicles—Using concepts from polymer chemistry to mimic biomembranes. Polymer.

[54] Jiang L., Vader P., Schiffelers R.M. (2017). Extracellular vesicles for nucleic acid delivery: Progress and prospects for safe RNA-based gene therapy. Gene Ther..

[55] Deming T.J. (2014). Preparation and development of block copolypeptide vesicles and hydrogels for biological and medical applications. WIREs Nanomed. Nanobiotechnol..

[56] van Hell A.J., Costa C.I.C.A., Flesch F.M., Sutter M., Jiskoot W., Crommelin D.J.A., Hennink W.E., Mastrobattista E. (2007). Self-Assembly of Recombinant Amphiphilic Oligopeptides into Vesicles. Biomacromolecules.

[57] Bellomo E.G., Wyrsta M.D., Pakstis L., Pochan D.J., Deming T.J. (2004). Stimuli-responsive polypeptide vesicles by conformation-specific assembly. Nat. Mater..

[58] Holowka E.P., Pochan D.J., Deming T.J. (2005). Charged Polypeptide Vesicles with Controllable Diameter. J. Am. Chem. Soc..

[59] Santoso S., Hwang W., Hartman H., Zhang S. (2002). Self-assembly of Surfactant-like Peptides with Variable Glycine Tails to Form Nanotubes and Nanovesicles. Nano Lett..

[60] Schuster T.B., de Bruyn Ouboter D., Bruns N., Meier W. (2011). Exploiting Dimerization of Purely Peptidic Amphiphiles to Form Vesicles. Small.

[61] Gudlur S., Sukthankar P., Gao J., Avila L.A., Hiromasa Y., Chen J., Iwamoto T., Tomich J.M. (2012). Peptide Nanovesicles Formed by the Self-Assembly of Branched Amphiphilic Peptides. PLoS ONE.

[62] Sunna A., Care A., Bergquist P.L. (2017). Peptides and Peptide-Based Biomaterials and Their Biomedical Applications.

[63] Spicer C.D., Jumeaux C., Gupta B., Stevens M.M. (2018). Peptide and protein nanoparticle conjugates: Versatile platforms for biomedical applications. Chem. Soc. Rev..

[64] Fominaya J., Bravo J., Rebollo A. (2015). Strategies to stabilize cell penetrating peptides for in vivo applications. Ther. Deliv..

[65] Goel R., Garg C., Gautam H.K., Sharma A.K., Kumar P., Gupta A. (2018). Fabrication of cationic nanostructures from short self-assembling amphiphilic mixed α/β-pentapeptide: Potential candidates for drug delivery, gene delivery, and antimicrobial applications. Int. J. Biol. Macromol..

[66] Eskandari S., Guerin T., Toth I., Stephenson R.J. (2017). Recent advances in self-assembled peptides: Implications for targeted drug delivery and vaccine engineering. Adv. Drug Deliv. Rev..

[67] Liang X., Shi B., Wang K., Fan M., Jiao D., Ao J., Song N., Wang C., Gu J., Li Z. (2016). Development of self-assembling peptide nanovesicle with bilayers for enhanced EGFR-targeted drug and gene delivery. Biomaterials.

[68] Wong S., Suk Shim M., Jik Kwon Y. (2014). Synthetically designed peptide-based biomaterials with stimuli-responsive and membrane-active properties for biomedical applications. J. Mater. Chem. B.

[69] Ding J., Xiao C., Zhuang X., He C., Chen X. (2012). Direct formation of cationic polypeptide vesicle as potential carrier for drug and gene. Mater. Lett..

[70] Iatrou H., Frielinghaus H., Hanski S., Ferderigos N., Ruokolainen J., Ikkala O., Richter D., Mays J., Hadjichristidis N. (2007). Architecturally Induced Multiresponsive Vesicles from Well-Defined Polypeptides. Formation of Gene Vehicles. Biomacromolecules.

[71] Zhang S., Marini D.M., Hwang W., Santoso S. (2002). Design of nanostructured biological materials through self-assembly of peptides and proteins. Curr. Opin. Chem. Biol..

[72] Han S., Cao S., Wang Y., Wang J., Xia D., Xu H., Zhao X., Lu J.R. (2011). Self-Assembly of Short Peptide Amphiphiles: The Cooperative Effect of Hydrophobic Interaction and Hydrogen Bonding. Chem. Eur. J..

[73] Zhang H., Park J., Jiang Y., Woodrow K.A. (2017). Rational Design of Charged Peptides that Self-Assemble into Robust Nanofibers as Immune-Functional Scaffolds. Acta Biomater..

[74] Hartgerink J.D., Beniash E., Stupp S.I. (2002). Peptide-amphiphile nanofibers: A versatile scaffold for the preparation of self-assembling materials. Proc. Natl. Acad. Sci. USA.

[75] Seroski D.T., Hudalla G.A., Sarmento B., das Neves J. (2018). Chapter 19-Self-Assembled Peptide and Protein Nanofibers for Biomedical Applications. Biomedical Applications of Functionalized Nanomaterials.

[76] Mazza M., Hadjidemetriou M., de Lázaro I., Bussy C., Kostarelos K. (2015). Peptide Nanofiber Complexes with siRNA for Deep Brain Gene Silencing by Stereotactic Neurosurgery. ACS Nano.

[77] Mello L.R., Hamley I.W., Castelletto V., Garcia B.B.M., Lourenço T.C., Vassiliades S.V., Alves W.A., Han S.W., Silva E.R. (2020). Self-assembly and intracellular delivery of DNA by a truncated fragment derived from the Trojan peptide Penetratin. Soft Matter.

[78] Bellat V., Ting R., Southard T.L., Vahdat L., Molina H., Fernandez J., Aras O., Stokol T., Law B. (2018). Functional Peptide Nanofibers with Unique Tumor Targeting and Enzyme-Induced Local Retention Properties. Adv. Funct. Mater..

[79] Zhang W., Lin D., Wang H., Li J., Nienhaus G.U., Su Z., Wei G., Shang L. (2017). Supramolecular Self-Assembly Bioinspired Synthesis of Luminescent Gold Nanocluster-Embedded Peptide Nanofibers for Temperature Sensing and Cellular Imaging. Bioconjug. Chem..

[80] Kim I., Han E.H., Ryu J., Min J.-Y., Ahn H., Chung Y.-H., Lee E. (2016). One-Dimensional Supramolecular Nanoplatforms for Theranostics Based on Co-Assembly of Peptide Amphiphiles. Biomacromolecules.

[81] Valéry C., Artzner F., Paternostre M. (2011). Peptide nanotubes: Molecular organisations, self-assembly mechanisms and applications. Soft Matter.

[82] Horne W.S., Stout C.D., Ghadiri M.R. (2003). A heterocyclic peptide nanotube. J. Am. Chem. Soc..

[83] Gao X., Matsui H. (2005). Peptide-Based Nanotubes and Their Applications in Bionanotechnology. Adv. Mater..

[84] Yuan C., Ji W., Xing R., Li J., Gazit E., Yan X. (2019). Hierarchically oriented organization in supramolecular peptide crystals. Nat. Rev. Chem..

[85] Fuhrhop J.-H., Koning J. (2007). Membranes and Molecular Assemblies: The Synkinetic Approach.

[86] Mandal D., Shirazi A.N., Parang K. (2014). Self-assembly of peptides to nanostructures. Org. Biomol. Chem..

[87] Ghadiri M.R., Granja J.R., Milligan R.A., McRee D.E., Khazanovich N. (1993). Self-assembling organic nanotubes based on a cyclic peptide architecture. Nature.

[88] Ghadiri M.R., Granja J.R., Buehler L.K. (1994). Artificial transmembrane ion channels from self-assembling peptide nanotubes. Nature.

[89] Hsieh W.-H., Chang S.-F., Chen H.-M., Chen J.-H., Liaw J. (2012). Oral Gene Delivery with cyclo-(d-Trp-Tyr) Peptide Nanotubes. Mol. Pharm..

[90] Vauthey S., Santoso S., Gong H., Watson N., Zhang S. (2002). Molecular self-assembly of surfactant-like peptides to form nanotubes and nanovesicles. Proc. Natl. Acad. Sci. USA.

[91] Wang M., Wang J., Zhou P., Deng J., Zhao Y., Sun Y., Yang W., Wang D., Li Z., Hu X. (2018). Nanoribbons self-assembled from short peptides demonstrate the formation of polar zippers between β-sheets. Nat. Commun..

[92] von Maltzahn G., Vauthey S., Santoso S., Zhang S. (2003). Positively Charged Surfactant-like Peptides Self-assemble into Nanostructures. Langmuir.

[93] Chapman R., Warr G.G., Perrier S., Jolliffe K.A. (2013). Water-Soluble and pH-Responsive Polymeric Nanotubes from Cyclic Peptide Templates. Chem. Eur. J..

[94] Larnaudie S.C., Brendel J.C., Romero-Canelón I., Sanchez-Cano C., Catrouillet S., Sanchis J., Coverdale J.P.C., Song J.-I., Habtemariam A., Sadler P.J. (2018). Cyclic Peptide–Polymer Nanotubes as Efficient and Highly Potent Drug Delivery Systems for Organometallic Anticancer Complexes. Biomacromolecules.

[95] Hsieh W.-H., Liaw J. (2019). Applications of cyclic peptide nanotubes (cPNTs). J. Food Drug Anal..

[96] Thapa R.K., Sullivan M.O. (2018). Gene delivery by peptide-assisted transport. Curr. Opin. Biomed. Eng..

[97] Raad M.D., Teunissen E.A., Mastrobattista E. (2014). Peptide vectors for gene delivery: From single peptides to multifunctional peptide nanocarriers. Nanomedicine.

[98] Schmidt-Wolf G.D., Schmidt-Wolf I.G.H. (2003). Non-viral and hybrid vectors in human gene therapy: An update. Trends Mol. Med..

[99] Ni R., Feng R., Chau Y. (2019). Synthetic Approaches for Nucleic Acid Delivery: Choosing the Right Carriers. Life.

[100] Murphy E.A., Waring A.J., Murphy J.C., Willson R.C., Longmuir K.J. (2001). Development of an effective gene delivery system: A study of complexes composed of a peptide-based amphiphilic DNA compaction agent and phospholipid. Nucleic Acids Res..

[101] Adami R.C., Collard W.T., Gupta S.A., Kwok K.Y., Bonadio J., Rice K.G. (1998). Stability of peptide-condensed plasmid DNA formulations. J. Pharm. Sci..

[102] Bloomfield V.A. (1991). Condensation of DNA by multivalent cations: Considerations on mechanism. Biopolymers.

[103] Mann A., Richa R., Ganguli M. (2008). DNA condensation by poly-l-lysine at the single molecule level: Role of DNA concentration and polymer length. J. Control. Release.

[104] Avila L.A., Aps L.R.M.M., Sukthankar P., Ploscariu N., Gudlur S., Šimo L., Szoszkiewicz R., Park Y., Lee S.Y., Iwamoto T. (2015). Branched Amphiphilic Cationic Oligopeptides Form Peptiplexes with DNA: A Study of Their Biophysical Properties and Transfection Efficiency. Mol. Pharm..

[105] Xu X., Yuan H., Chang J., He B., Gu Z. (2012). Cooperative Hierarchical Self-Assembly of Peptide Dendrimers and Linear Polypeptides into Nanoarchitectures Mimicking Viral Capsids. Angew. Chem. Int. Ed..

[106] Koo H.-B., Kang H.-S., Lee Y. (2009). Analysis of the Relationship between the Molecular Weight and Transfection Efficiency/Cytotoxicity of Poly-l-arginine on a Mammalian Cell Line. Bull. Korean Chem. Soc..

[107] Rice K.G., Wadhwa M.S. (2002). Cationic Peptides, Cys-Trp-(LYS)n, for Gene Delivery. U.S. Patent.

[108] Wolfert M.A., Seymour L.W. (1998). Chloroquine and amphipathic peptide helices show synergistic transfection in vitro. Gene Ther..

[109] Futaki S., Nakase I. (2017). Cell-Surface Interactions on Arginine-Rich Cell-Penetrating Peptides Allow for Multiplex Modes of Internalization. Acc. Chem. Res..

[110] Zhou Y., Han S., Liang Z., Zhao M., Liu G., Wu J. (2020). Progress in arginine-based gene delivery systems. J. Mater. Chem. B.

[111] Bennett R., Yakkundi A., McKeen H.D., McClements L., McKeogh T.J., McCrudden C.M., Arthur K., Robson T., McCarthy H.O. (2015). RALA-mediated delivery of FKBPL nucleic acid therapeutics. Nanomedicine.

[112] Ali A.A., McCrudden C.M., McCaffrey J., McBride J.W., Cole G., Dunne N.J., Robson T., Kissenpfennig A., Donnelly R.F., McCarthy H.O. (2017). DNA vaccination for cervical cancer; a novel technology platform of RALA mediated gene delivery via polymeric microneedles. Nanomed. Nanotechnol. Biol. Med..

[113] McCrudden C.M., McBride J.W., McCaffrey J., McErlean E.M., Dunne N.J., Kett V.L., Coulter J.A., Robson T., McCarthy H.O. (2018). Gene therapy with RALA/iNOS composite nanoparticles significantly enhances survival in a model of metastatic prostate cancer. Cancer Nanotechnol..

[114] Cole G., Ali A.A., McCrudden C.M., McBride J.W., McCaffrey J., Robson T., Kett V.L., Dunne N.J., Donnelly R.F., McCarthy H.O. (2018). DNA vaccination for cervical cancer: Strategic optimisation of RALA mediated gene delivery from a biodegradable microneedle system. Eur. J. Pharm. Biopharm..

[115] Midoux P., Pichon C., Yaouanc J.-J., Jaffrès P.-A. (2009). Chemical vectors for gene delivery: A current review on polymers, peptides and lipids containing histidine or imidazole as nucleic acids carriers. Br. J. Pharmacol..

[116] Aoki Y., Hosaka S., Kawa S., Kiyosawa K. (2001). Potential tumor-targeting peptide vector of histidylated oligolysine conjugated to a tumor-homing RGD motif. Cancer Gene Ther..

[117] Leng Q., Mixson A.J. (2005). Modified branched peptides with a histidine-rich tail enhance in vitro gene transfection. Nucleic Acids Res..

[118] El-Sayed N.S., Miyake T., Shirazi A.N., Park S.E., Clark J., Buchholz S., Parang K., Tiwari R. (2018). Design, Synthesis, and Evaluation of Homochiral Peptides Containing Arginine and Histidine as Molecular Transporters. Mol. J. Synth. Chem. Nat. Prod. Chem..

[119] Tang Q., Cao B., Wu H., Cheng G. (2012). Selective Gene Delivery to Cancer Cells Using an Integrated Cationic Amphiphilic Peptide. Langmuir.

[120] Zhu Z., Tian D., Gao P., Wang K., Li Y., Shu X., Zhu J., Zhao Q. (2018). Cell-Penetrating Peptides Transport Noncovalently Linked Thermally Activated Delayed Fluorescence Nanoparticles for Time-Resolved Luminescence Imaging. J. Am. Chem. Soc..

[121] Zou Q., Abbas M., Zhao L., Li S., Shen G., Yan X. (2017). Biological Photothermal Nanodots Based on Self-Assembly of Peptide–Porphyrin Conjugates for Antitumor Therapy. J. Am. Chem. Soc..

[122] Whitehead K.A., Langer R., Anderson D.G. (2009). Knocking down barriers: Advances in siRNA delivery. Nat. Rev. Drug Discov..

[123] Khalil I.A., Harashima H. (2018). An efficient PEGylated gene delivery system with improved targeting: Synergism between octaarginine and a fusogenic peptide. Int. J. Pharm..

[124] Mishra S., Webster P., Davis M.E. (2004). PEGylation significantly affects cellular uptake and intracellular trafficking of non-viral gene delivery particles. Eur. J. Cell Biol..

[125] Hamley I.W. (2014). PEG—Peptide Conjugates. Biomacromolecules.

[126] Osman G., Rodriguez J., Chan S.Y., Chisholm J., Duncan G., Kim N., Tatler A.L., Shakesheff K.M., Hanes J., Suk J.S. (2018). PEGylated enhanced cell penetrating peptide nanoparticles for lung gene therapy. J. Control. Release.

[127] Alhakamy N.A., Nigatu A.S., Berkland C.J., Ramsey J.D. (2013). Noncovalently associated-penetrating peptides for gene delivery applications. Ther. Deliv..

[128] Taylor R.E., Zahid M. (2020). Cell Penetrating Peptides, Novel Vectors for Gene Therapy. Pharmaceutics.

[129] de Figueiredo I.R., Freire J.M., Flores L., Veiga A.S., Castanho M.A.R.B. (2014). Cell-penetrating peptides: A tool for effective delivery in gene-targeted therapies. IUBMB Life.

[130] Pack D.W., Hoffman A.S., Pun S., Stayton P.S. (2005). Design and development of polymers for gene delivery. Nat. Rev. Drug Discov..

[131] Mandal H., Katiyar S.S., Swami R., Kushwah V., Katare P.B., Kumar Meka A., Banerjee S.K., Popat A., Jain S. (2018). ε-Poly-l-Lysine/plasmid DNA nanoplexes for efficient gene delivery in vivo. Int. J. Pharm..

[132] Walsh D.P., Raftery R.M., Castaño I.M., Murphy R., Cavanagh B., Heise A., O’Brien F.J., Cryan S.-A. (2019). Transfection of autologous host cells in vivo using gene activated collagen scaffolds incorporating star-polypeptides. J. Control. Release.

[133] Ziady A.-G., Gedeon C.R., Miller T., Quan W., Payne J.M., Hyatt S.L., Fink T.L., Muhammad O., Oette S., Kowalczyk T. (2003). Transfection of airway epithelium by stable PEGylated poly-l-lysine DNA nanoparticles in vivo. Mol. Ther..

[134] Wang G., Gao X., Gu G., Shao Z., Li M., Wang P., Yang J., Cai X., Li Y. (2017). Polyethylene glycol–poly(ε-benzyloxycarbonyl-l-lysine)-conjugated VEGF siRNA for antiangiogenic gene therapy in hepatocellular carcinoma. Int. J. Nanomed..

[135] Midoux P., Breuzard G., Gomez J., Pichon C. (2008). Polymer-Based Gene Delivery: A Current Review on the Uptake and Intracellular Trafficking of Polyplexes. Curr. Gene Ther..

[136] van Rossenberg S., van Keulen A., Drijfhout J.-W., Vasto S., Koerten H.K., Spies F., van ’t Noordende J., van Berkel T., Biessen E.A.L. (2004). Stable polyplexes based on arginine-containing oligopeptides for in vivo gene delivery. Gene Ther..

[137] Loughran S.P., McCrudden C.M., McCarthy H.O. (2015). Designer peptide delivery systems for gene therapy. Eur. J. Nanomed..

[138] Kim H.H., Choi H.S., Yang J.M., Shin S. (2007). Characterization of gene delivery in vitro and in vivo by the arginine peptide system. Int. J. Pharm..

[139] Ul Ain Q., Chung H., Chung J.Y., Choi J.-H., Kim Y.-H. (2017). Amelioration of atherosclerotic inflammation and plaques via endothelial adrenoceptor-targeted eNOS gene delivery using redox-sensitive polymer bearing l-arginine. J. Control. Release.

[140] Hyun H., Won Y.-W., Kim K.-M., Lee J., Lee M., Kim Y.-H. (2010). Therapeutic effects of a reducible poly (oligo-d-arginine) carrier with the heme oxygenase-1 gene in the treatment of hypoxic-ischemic brain injury. Biomaterials.

[141] Won Y.-W., Kim K.-M., An S.S., Lee M., Ha Y., Kim Y.-H. (2011). Suicide gene therapy using reducible poly (oligo-d-arginine) for the treatment of spinal cord tumors. Biomaterials.

[142] Woo J., Bae S.-H., Kim B., Park J.S., Jung S., Lee M., Kim Y.-H., Choi D. (2015). Cardiac Usage of Reducible Poly(oligo-D-arginine) As a Gene Carrier for Vascular Endothelial Growth Factor Expression. PLoS ONE.

[143] Won Y.-W., Kim H.A., Lee M., Kim Y.-H. (2010). Reducible Poly(oligo-d-arginine) for Enhanced Gene Expression in Mouse Lung by Intratracheal Injection. Mol. Ther..

[144] Kesharwani P., Iyer A.K. (2015). Recent advances in dendrimer-based nanovectors for tumor-targeted drug and gene delivery. Drug Discov. Today.

[145] Torchilin V.P. (2008). Cell penetrating peptide-modified pharmaceutical nanocarriers for intracellular drug and gene delivery. Pept. Sci..

[146] Luo K., Li C., Li L., She W., Wang G., Gu Z. (2012). Arginine functionalized peptide dendrimers as potential gene delivery vehicles. Biomaterials.

[147] Kozhikhova K.V., Andreev S.M., Shilovskiy I.P., Timofeeva A.V., Gaisina A.R., Shatilov A.A., Turetskiy E.A., Andreev I.M., Smirnov V.V., Dvornikov A.S. (2018). A novel peptide dendrimer LTP efficiently facilitates transfection of mammalian cells. Org. Biomol. Chem..

[148] Yoo J., Lee D., Gujrati V., Rejinold N.S., Lekshmi K.M., Uthaman S., Jeong C., Park I.-K., Jon S., Kim Y.-C. (2017). Bioreducible branched poly(modified nona-arginine) cell-penetrating peptide as a novel gene delivery platform. J. Control. Release.

[149] Tang M., Dong H., Li Y., Ren T. (2016). Harnessing the PEG-cleavable strategy to balance cytotoxicity, intracellular release and the therapeutic effect of dendrigraft poly-L-lysine for cancer gene therapy. J. Mater. Chem. B.

[150] Johnson L.N., Cashman S.M., Kumar-Singh R. (2008). Cell-penetrating Peptide for Enhanced Delivery of Nucleic Acids and Drugs to Ocular Tissues Including Retina and Cornea. Mol. Ther..

[151] Lehto T., Simonson O.E., Mäger I., Ezzat K., Sork H., Copolovici D.-M., Viola J.R., Zaghloul E.M., Lundin P., Moreno P.M. (2011). A Peptide-based Vector for Efficient Gene Transfer In Vitro and In Vivo. Mol. Ther..

[152] EL Andaloussi S., Lehto T., Mäger I., Rosenthal-Aizman K., Oprea I.I., Simonson O.E., Sork H., Ezzat K., Copolovici D.M., Kurrikoff K. (2011). Design of a peptide-based vector, PepFect6, for efficient delivery of siRNA in cell culture and systemically in vivo. Nucleic Acids Res..

[153] Aldrian G., Vaissière A., Konate K., Seisel Q., Vivès E., Fernandez F., Viguier V., Genevois C., Couillaud F., Démèné H. (2017). PEGylation rate influences peptide-based nanoparticles mediated siRNA delivery in vitro and in vivo. J. Control. Release.

[154] Rittner K., Benavente A., Bompard-Sorlet A., Heitz F., Divita G., Brasseur R., Jacobs E. (2002). New Basic Membrane-Destabilizing Peptides for Plasmid-Based Gene Delivery in Vitro and in Vivo. Mol. Ther..

[155] Liu Y., Song Z., Zheng N., Nagasaka K., Yin L., Cheng J. (2018). Systemic siRNA delivery to tumors by cell-penetrating α-helical polypeptide-based metastable nanoparticles. Nanoscale.

[156] He H., Zheng N., Song Z., Kim K.H., Yao C., Zhang R., Zhang C., Huang Y., Uckun F.M., Cheng J. (2016). Suppression of Hepatic Inflammation via Systemic siRNA Delivery by Membrane-Disruptive and Endosomolytic Helical Polypeptide Hybrid Nanoparticles. ACS Nano.

[157] Smits F.C.M., Buddingh B.C., van Eldijk M.B., van Hest J.C.M. (2015). Elastin-Like Polypeptide Based Nanoparticles: Design Rationale Toward Nanomedicine. Macromol. Biosci..

[158] Primiano T., Chang B.-D., Heidel J.D. (2014). Abstract LB-103: L1CAM-targeted delivery of siRNA using elastin-like polypeptide (ELP) nanoparticles inhibits the growth of human tumors implanted in mice. Cancer Res..

[159] Bidwell G.L., Raucher D. (2010). Cell penetrating elastin-like polypeptides for therapeutic peptide delivery. Adv. Drug Deliv. Rev..

[160] Yi A., Sim D., Lee Y.-J., Sarangthem V., Park R.-W. (2020). Development of elastin-like polypeptide for targeted specific gene delivery in vivo. J. Nanobiotechnol..

[161] Xiang S., Tong H., Shi Q., Fernandes J.C., Jin T., Dai K., Zhang X. (2012). Uptake mechanisms of non-viral gene delivery. J. Control. Release.

[162] Varkouhi A.K., Scholte M., Storm G., Haisma H.J. (2011). Endosomal escape pathways for delivery of biologicals. J. Control. Release.

[163] Meng Z., Luan L., Kang Z., Feng S., Meng Q., Liu K. (2016). Histidine-enriched multifunctional peptide vectors with enhanced cellular uptake and endosomal escape for gene delivery. J. Mater. Chem. B.

[164] Liu J., Guo N., Gao C., Liu N., Zheng X., Tan Y., Lei J., Hao Y., Chen L., Zhang X. (2019). Effective Gene Silencing Mediated by Polypeptide Nanoparticles LAH4-L1-siMDR1 in Multi-Drug Resistant Human Breast Cancer. J. Biomed. Nanotechnol..

[165] Asayama S., Sekine T., Kawakami H., Nagaoka S. (2007). Design of Aminated Poly(1-vinylimidazole) for a New pH-Sensitive Polycation To Enhance Cell-Specific Gene Delivery. Bioconjug. Chem..

[166] Perche F., Gosset D., Mével M., Miramon M.-L., Yaouanc J.-J., Pichon C., Benvegnu T., Jaffrès P.-A., Midoux P. (2011). Selective gene delivery in dendritic cells with mannosylated and histidylated lipopolyplexes. J. Drug Target..

[167] Perche F., Benvegnu T., Berchel M., Lebegue L., Pichon C., Jaffrès P.-A., Midoux P. (2011). Enhancement of dendritic cells transfection in vivo and of vaccination against B16F10 melanoma with mannosylated histidylated lipopolyplexes loaded with tumor antigen messenger RNA. Nanomed. Nanotechnol. Biol. Med..

[168] Perche F., Lambert O., Berchel M., Jaffrès P.-A., Pichon C., Midoux P. (2012). Gene transfer by histidylated lipopolyplexes: A dehydration method allowing preservation of their physicochemical parameters and transfection efficiency. Int. J. Pharm..

[169] Zhu H., Dong C., Dong H., Ren T., Wen X., Su J., Li Y. (2014). Cleavable PEGylation and Hydrophobic Histidylation of Polylysine for siRNA Delivery and Tumor Gene Therapy. ACS Appl. Mater. Interfaces.

[170] Zhou J., Zhao Y., Simonenko V., Xu J.J., Liu K., Wang D., Shi J., Zhong T., Zhang L., Zeng L. (2017). Simultaneous silencing of TGF-β1 and COX-2 reduces human skin hypertrophic scar through activation of fibroblast apoptosis. Oncotarget.

[171] Lu P.Y., Ansari A., Guan P.J., Xu J.J., Simonenko V., Zhong T. (2018). Gemcitabine Derivatives for Cancer Therapy. U.S. Patent.

[172] Yao C., Liu J., Wu X., Tai Z., Gao Y., Zhu Q., Li J., Zhang L., Hu C., Gu F. (2016). Reducible self-assembling cationic polypeptide-based micelles mediate co-delivery of doxorubicin and microRNA-34a for androgen-independent prostate cancer therapy. J. Control. Release.

[173] Tai Z., Wang X., Tian J., Gao Y., Zhang L., Yao C., Wu X., Zhang W., Zhu Q., Gao S. (2015). Biodegradable Stearylated Peptide with Internal Disulfide Bonds for Efficient Delivery of siRNA In Vitro and In Vivo. Biomacromolecules.

[174] Cai X., Jin R., Wang J., Yue D., Jiang Q., Wu Y., Gu Z. (2016). Bioreducible Fluorinated Peptide Dendrimers Capable of Circumventing Various Physiological Barriers for Highly Efficient and Safe Gene Delivery. ACS Appl. Mater. Interfaces.

[175] Meikle S.R., Kench P., Kassiou M., Banati R.B. (2005). Small animal SPECT and its place in the matrix of molecular imaging technologies. Phys. Med. Biol..

[176] Que E.L., Chang C.J. (2009). Responsive magnetic resonance imaging contrast agents as chemical sensors for metals in biology and medicine. Chem. Soc. Rev..

[177] Sun X., Cai W., Chen X. (2015). Positron Emission Tomography Imaging Using Radiolabeled Inorganic Nanomaterials. Acc. Chem. Res..

[178] Ferrara K.W., Borden M.A., Zhang H. (2009). Lipid-Shelled Vehicles: Engineering for Ultrasound Molecular Imaging and Drug Delivery. Acc. Chem. Res..

[179] Giepmans B.N.G., Adams S.R., Ellisman M.H., Tsien R.Y. (2006). The Fluorescent Toolbox for Assessing Protein Location and Function. Science.

[180] Lee D.-E., Koo H., Sun I.-C., Ryu J.H., Kim K., Kwon I.C. (2012). Multifunctional nanoparticles for multimodal imaging and theragnosis. Chem. Soc. Rev..

[181] Bhushan S., Anandasabapathy S., Petrova E. (2019). Photoacoustic Imaging in Gastroenterology: Advances and Needs. Photoacoust. Imaging-Princ. Adv. Appl..

[182] Lin C., Luciani A., Itti E., Haioun C., Rahmouni A. (2007). Whole body MRI and PET/CT in haematological malignancies. Cancer Imaging.

[183] Genc A., Ryk M., Suwała M., Żurakowska T., Kosiak W. (2016). Ultrasound imaging in the general practitioner’s office—A literature review. J. Ultrason..

[184] Ni M., Zhuo S. (2019). Applications of self-assembling ultrashort peptides in bionanotechnology. RSC Adv..

[185] Pernot M., Vanderesse R., Frochot C., Guillemin F., Barberi-Heyob M. (2011). Stability of peptides and therapeutic success in cancer. Expert Opin. Drug Metab. Toxicol..

[186] Ma J., Dong H., Zhu H., Li C., Li Y., Shi D. (2016). Deposition of gadolinium onto the shell structure of micelles for integrated magnetic resonance imaging and robust drug delivery systems. J. Mater. Chem. B.

[187] Wu L., Fang S., Shi S., Deng J., Liu B., Cai L. (2013). Hybrid Polypeptide Micelles Loading Indocyanine Green for Tumor Imaging and Photothermal Effect Study. Biomacromolecules.

[188] An H.-W., Hou D., Zheng R., Wang M.-D., Zeng X.-Z., Xiao W.-Y., Yan T.-D., Wang J.-Q., Zhao C.-H., Cheng L.-M. (2020). A Near-Infrared Peptide Probe with Tumor-Specific Excretion-Retarded Effect for Image-Guided Surgery of Renal Cell Carcinoma. ACS Nano.

[189] Zhang D., Qi G.-B., Zhao Y.-X., Qiao S.-L., Yang C., Wang H. (2015). In Situ Formation of Nanofibers from Purpurin18-Peptide Conjugates and the Assembly Induced Retention Effect in Tumor Sites. Adv. Mater..

[190] Liu Y., Bhattarai P., Dai Z., Chen X. (2019). Photothermal therapy and photoacoustic imaging via nanotheranostics in fighting cancer. Chem. Soc. Rev..

[191] Nie L., Chen X. (2014). Structural and functional photoacoustic molecular tomography aided by emerging contrast agents. Chem. Soc. Rev..

[192] Jeena M.T., Jeong K., Go E.M., Cho Y., Lee S., Jin S., Hwang S.-W., Jang J.H., Kang C.S., Bang W.-Y. (2019). Heterochiral Assembly of Amphiphilic Peptides Inside the Mitochondria for Supramolecular Cancer Therapeutics. ACS Nano.

[193] Yang P.-P., Luo Q., Qi G.-B., Gao Y.-J., Li B.-N., Zhang J.-P., Wang L., Wang H. (2017). Host Materials Transformable in Tumor Microenvironment for Homing Theranostics. Adv. Mater..

[194] Qian Y., Wang W., Wang Z., Jia X., Han Q., Rostami I., Wang Y., Hu Z. (2018). pH-Triggered Peptide Self-Assembly for Targeting Imaging and Therapy toward Angiogenesis with Enhanced Signals. ACS Appl. Mater. Interfaces.

[195] Zhang J., Mu Y.-L., Ma Z.-Y., Han K., Han H.-Y. (2018). Tumor-triggered transformation of chimeric peptide for dual-stage-amplified magnetic resonance imaging and precise photodynamic therapy. Biomaterials.

[196] Brendel J.C., Sanchis J., Catrouillet S., Czuba E., Chen M.Z., Long B.M., Nowell C., Johnston A., Jolliffe K.A., Perrier S. (2018). Secondary Self-Assembly of Supramolecular Nanotubes into Tubisomes and Their Activity on Cells. Angew. Chem. Int. Ed..

[197] Mansfield E.D.H., Hartlieb M., Catrouillet S., Rho J.Y., Larnaudie S.C., Rogers S.E., Sanchis J., Brendel J.C., Perrier S. (2018). Systematic study of the structural parameters affecting the self-assembly of cyclic peptide–poly(ethylene glycol) conjugates. Soft Matter.

[198] Hourani R., Zhang C., van der Weegen R., Ruiz L., Li C., Keten S., Helms B.A., Xu T. (2011). Processable Cyclic Peptide Nanotubes with Tunable Interiors. J. Am. Chem. Soc..

[199] Catrouillet S., Brendel J.C., Larnaudie S., Barlow T., Jolliffe K.A., Perrier S. (2016). Tunable Length of Cyclic Peptide–Polymer Conjugate Self-Assemblies in Water. ACS Macro Lett..

[200] Chen J., Zhang B., Xia F., Xie Y., Jiang S., Su R., Lu Y., Wu W. (2016). Transmembrane delivery of anticancer drugs through self-assembly of cyclic peptide nanotubes. Nanoscale.

[201] Fan Z., Chang Y., Cui C., Sun L., Wang D.H., Pan Z., Zhang M. (2018). Near infrared fluorescent peptide nanoparticles for enhancing esophageal cancer therapeutic efficacy. Nat. Commun..

[202] Guo H., Song S., Dai T., Sun K., Zhou G., Li M., Mann S., Dou H. (2020). Near-Infrared Fluorescent and Magnetic Resonance Dual-Imaging Coacervate Nanoprobes for Trypsin Mapping and Targeted Payload Delivery of Malignant Tumors. ACS Appl. Mater. Interfaces.

[203] Yan R., Hu Y., Liu F., Wei S., Fang D., Shuhendler A.J., Liu H., Chen H.-Y., Ye D. (2019). Activatable NIR Fluorescence/MRI Bimodal Probes for in Vivo Imaging by Enzyme-Mediated Fluorogenic Reaction and Self-Assembly. J. Am. Chem. Soc..

[204] Sun X., Li Y., Liu T., Li Z., Zhang X., Chen X. (2017). Peptide-based imaging agents for cancer detection. Adv. Drug Deliv. Rev..

[205] Phillips E., Penate-Medina O., Zanzonico P.B., Carvajal R.D., Mohan P., Ye Y., Humm J., Gönen M., Kalaigian H., Schöder H. (2014). Clinical translation of an ultrasmall inorganic optical-PET imaging nanoparticle probe. Sci. Transl. Med..

